# Applying an intersectional framework to characterize chronic pain disparities one year following traumatic spinal cord injury

**DOI:** 10.1016/j.jpain.2026.106371

**Published:** 2026-07-06

**Authors:** Joel Fundaun, Titilola Falasinnu, Dario Pfyffer, Troy C. Dildine, Melissa Kolski, Allen W. Heinemann, Suzanne Tharin, Andrew C. Smith, Benjamin Dirlikov, Sean Mackey, James Crew, Kenneth A. Weber

**Affiliations:** a Department of Anesthesiology, Perioperative, and Pain Medicine, Stanford University School of Medicine, Stanford, CA, USA; b Department of Medicine, Stanford University School of Medicine, Stanford, CA, USA; c Department of Physical Therapy and Human Movement Sciences, Northwestern University, Feinberg School of Medicine, Chicago, IL, USA; d Musculoskeletal Outpatient Department, Shirley Ryan AbilityLab, Chicago, IL, USA; e Department of Physical Medicine and Rehabilitation, Feinberg School of Medicine, Northwestern University, Chicago, IL, USA; f Center for Rehabilitation Outcomes Research, Shirley Ryan AbilityLab, Chicago, IL, USA; g Department of Neurosurgery, Stanford University Medical Center, Stanford, CA, USA; h Division of Neurosurgery, VA, Palo Alto, CA, USA; i Physical Therapy Program, Department of Physical Medicine and Rehabilitation, School of Medicine, University of Colorado, Aurora, CO, USA; j Rehabilitation Research Center, Santa Clara Valley Medical Center, San Jose, CA, USA; k Department of Physical Medicine and Rehabilitation, Santa Clara Valley Medical Center, San Jose, CA, USA

**Keywords:** Spinal cord injury, Chronic pain, Social determinants of health, Health inequities, Mediation

## Abstract

Chronic pain affects nearly 70% of individuals following traumatic spinal cord injury (SCI) and is frequently reported as a disabling and undertreated complication. While disparities in SCI recovery are well-documented, evidence in chronic pain remains limited, with prior studies involving smaller cohorts and evaluating isolated social determinants rather than their intersectional contributions. We conducted a retrospective cohort study using the US Spinal Cord Injury Model Systems database, including 3514 adults with traumatic SCI. Exposures were race/ethnicity (non-Hispanic White, non-Hispanic Black), annual income (<$25,000 vs ≥$25,000), and education (high school or less vs higher). Outcomes were pain severity (0–10 numeric rating) and pain interference (SF-12; severe[3–4] vs minimal/moderate[0–2]). Models adjusted for demographic and injury characteristics. Intersectional analyses quantified the combined effects of multiple social disadvantages, and causal mediation analyses estimated average causal mediation effects (ACME). Non-Hispanic Black, lower-income, and lower-education participants had higher pain severity and more frequent severe pain interference than their counterparts (all p<0.0001). Non-Hispanic Black participants, low-income, low-education participants had +2.43 points (95%CI 2.06–2.79) higher pain severity and 2.96-fold (95%CI 2.15–4.07) higher odds of severe pain interference than non-Hispanic White, higher-income, higher-education participants (q<0.0001). Mobility scores, days out-of-the-house, private health insurance, and perceived health status significantly mediated disparities in pain severity (ACME 0.158–0.312) and pain interference (ACME 0.017–0.043; all q<0.001). Chronic pain after traumatic SCI disproportionately affects socially disadvantaged groups. These findings highlight intersectional disparities and identify modifiable mediators as targets for interventions to reduce inequitable pain outcomes following traumatic SCI.

**Perspective:**

This retrospective multicenter cohort study quantified intersectional disparities in chronic pain one year following spinal cord injury and characterized modifiable individual and structural mediators that account for these inequities.

## Introduction

Chronic pain affects nearly 70% of all individuals following traumatic spinal cord injury (SCI), impairing recovery, and reducing quality of life.^1^ While individuals with chronic traumatic SCI consistently report improved pain management as a high priority,^2^ current strategies demonstrate only moderate efficacy.^3^ Approximately 18% of patients develop substance use behaviors to cope with chronic pain,^4^ reinforcing the need to recognize risk factors to more effectively evaluate and treat chronic pain following SCI.

The expansive burden of chronic pain has been shown to disproportionally affect individuals with historically documented social disadvantages,^5^ which here is defined as non-health related identities and social factors (e.g., low socioeconomic status) that decrease one’s access to resources and increase one’s risks for negative social stressors (e.g., discrimination) and illness. Across chronic pain conditions, minoritized race/ethnicity and lower socioeconomic status have been associated with higher pain severity, duration, and disability compared to their more advantaged counter groups.^6^ In SCI, minoritized race and ethnicity, lower income, and lower education have been associated with reduced rates of acute surgical decompression, increased hospital complications and days in hospital, reduced likelihood of inpatient rehabilitation services, and increased mortality.^7-10^ In the US, traumatic SCI also occurs at disproportionately higher rates among individuals identifying as Black, compounding the effects of structural racism and limited healthcare access present in SCI.^11^

Despite well-established inequities identified separately in both chronic pain and traumatic SCI, research evaluating disparities in individuals with chronic pain following traumatic SCI include small sample sizes with simple analytical frameworks. Moreover, most prior studies evaluated individual social indicators, leaving intersectional effects (i.e., combined contributions) of multiple social disadvantages on chronic pain outcomes unexplored. Intersectionality here refers to the combined and interdependent effects of social disadvantage on chronic pain outcomes.^12^ In addition, little is known about the mechanisms through which social disadvantage may influence pain outcomes following traumatic SCI.^13^ Previous mediation analyses in SCI have suggested that community reintegration and socioeconomic factors mediate functional recovery after SCI.^14,15^ Evaluating these relationships with respect to chronic pain could enable more equitable and effective treatment strategies to reduce the disproportionate burden of chronic pain following traumatic SCI.

The overall aim of this study was to quantify the individual and intersectional effects of race, income, and education on pain severity and pain interference one-year following traumatic SCI using the US Spinal Cord Injury Model Systems (SCIMS) database, the largest global repository of longitudinal traumatic SCI outcomes. We further sought to quantify the relative contributions of these social indicators and identify potential mediating effects of acute and one-year variables on pain outcomes. By integrating intersectional modeling with mediation analyses in a large national SCI cohort, this study moves beyond identifying disparities to quantify the mediating pathways through which social disadvantage influences chronic pain post-SCI.

## Materials and methods

### Study design and participant selection

We conducted a multicenter retrospective cohort study in participants with traumatic SCI using the publicly available SCIMS database, the largest global repository of longitudinal traumatic SCI outcomes.^11^ The SCIMS database prospectively enrolls participants after acute traumatic SCI from 18 designated centers in the US. This nationally representative dataset includes demographic and injury characteristics, medical, functional, psychosocial, employment, and survival outcomes. Data are collected at multiple acute and longitudinal follow-up timepoints, beginning with acute hospital admission, rehabilitation admission and discharge, and one-year post-injury. Participants are then followed at five-year intervals (i.e., 5, 10, 15 years post-injury). Full details of participant definitions, SCIMS eligibility, data collection, and addition of new variables over time have been previously published.^11,16^ As this study used only the publicly available, de-identified SCIMS database, additional IRB approval and informed consent were not required.

We included traumatic SCI participants from the de-identified SCIMS database collected from cohort inception to September 2021 with complete datasets for age at injury, sex, body mass index (BMI, calculated from recorded height and weight), neurological level of injury (rehabilitation discharge), American Spinal Injury Association Impairment Scale (AIS) grade at rehabilitation discharge, which is a standardized neurological assessment to grade the severity of SCI, injury etiology, race and ethnicity, annual household income (acute admission), and educational attainment (acute admission). As our primary objective was to evaluate differences in chronic pain, participants reporting pain scores of zero were excluded. Variables associated with neurological status were selected from rehabilitation discharge, as this timepoint represents a more neurologically stable phase used previously with the least amount of missing data.^17^ We included participants who reported annual household income from the acute assessment (versus one-year follow-up) to avoid confounding by employment status changes following injury.

Participants were excluded if they had incomplete baseline demographics and injury characteristics, or missing pain severity measures at one-year follow-up. As previous studies identified lower follow-up rates for SCIMS participants from socially disadvantaged backgrounds,^18^ we evaluated patterns of participant attrition and missing data to assess potential selection bias. Previous analyses of the SCIMS database demonstrated higher rates of loss to follow-up after acute traumatic SCI for minoritized racial/ethnic participants, those with lower education, unemployment, violent injury etiology, and lack of health insurance.^18^ To evaluate potential bias within our defined SCIMS dataset, we compared demographic and socioeconomic characteristics between our included study participants and those excluded but still within the SCIMS database. We compared participants included versus those excluded using two methods: 1) All SCIMS participants meeting study baseline eligibility, regardless of missing variables; 2) participants limited by our study cohort selection criteria. We calculated an overall association between each variable and inclusion status using Pearson’s χ^2^ test (two-sided α=0.05) and effect sizes. Additional details are included in Supplemental Tables 1-2 and Supplemental Figures 1-2.

### Pain outcomes

Pain severity was assessed one-year post-injury using a numeric rating scale, where participants were asked: "Using a 0–10 scale with 10 being pain so severe you could not stand it and 0 being no pain, what has been the usual level of pain over the past 4 weeks?" Pain interference with daily activities was measured using the SF-12 pain interference item asking "During the past 4 weeks, how much did pain interfere with your normal work including both work outside the home and housework (or usual activities)?"^19^ SF-12 response options included: Not at all (0), A little bit (1), Moderately (2), Quite a bit (3), and Extremely (4). For participants who did not work, the question focused on interference with usual activities as determined by the participant. We dichotomized pain interference as low/moderate (scores 0–2) versus severe (scores 3–4), aligned with conservative classifications for high impact chronic pain based on the Graded Chronic Pain Scale-Revised.^20^

### Sociodemographic factors

Race was categorized using the SCIMS self-reported definitions of White, Black, American Indian/Alaska Native, Asian/Pacific Islander, or Other/Multiracial. Ethnicity was classified as Hispanic or non-Hispanic. We created combined race-ethnicity classifications for all assessments and note that the SCIMS categories differ slightly from US Census categories,^21^ with Asian/Pacific Islander typically separated as ‘Asian American’ and ‘Pacific Islander or Native Hawaiian.’ The term “race” is used throughout, as our primary analyses focused on non-Hispanic White and non-Hispanic Black participants, which were the only groups representing at least 10% of the cohort. All other race and ethnicity combinations were below this threshold and were analyzed separately and reported in Supplemental Material. Self-reported annual household income at acute admission included the combined income of the participant and family members living in the same household. Income was dichotomized as lower income (≤$25,000 per year) versus higher income (>$25,000 per year).^22^ The level of educational attainment at the time of injury was self-reported at acute admission as the highest level of formal education completed. Education was dichotomized as lower (high school diploma or less) versus higher (any post-secondary education, including associate’s, bachelor’s, or graduate degrees). To assess differences in outcomes based on definitions of socioeconomic status, we performed sensitivity analyses using alternative thresholds. For yearly family income, we compared our primary classification (<$25,000 vs. ≥$25,000) with a higher cut-off (<$50,000 vs. ≥$50,000). For participants’ education level, we compared our primary definition (high school or less vs. any college) with a classification including a higher baseline educational level (up to bachelor's degree vs. graduate degree or other).

### Statistical analysis

All analyses were conducted in R version 4.4.1. Participant characteristics are reported using means and standard deviations for continuous variables and frequencies and percentages for categorical variables. We estimated differences in pain severity and pain interference across intersecting sociodemographic groups defined by race and ethnicity, income, and education. We modeled pain severity using multivariable linear regression, and pain interference using logistic regression (severe vs. minimal/moderate), each adjusted for potential confounders including age at injury, sex, BMI, neurological level of injury (cervical, thoracic, lumbar, and sacral), AIS grade (A-E), and injury etiology. We evaluated adjusted group means (continuous pain severity) and probabilities/odds ratios (dichotomized pain interference) using estimated marginal means with 95% confidence intervals (CI) for each model. Exploratory analyses evaluated differences in continuous pain severity scores within each dichotomized pain interference group using Mann-Whitney U and Holm’s method for multiple testing performed separately within the single-level pain severity, interference, and combined severity-interference analyses.

Intersectional analyses examined two- and three-way interactions between the intersections of race with income and/or education using interaction factors (race×income, race×education, income×education, and race×income×education). The most socially advantaged group (non-Hispanic White participants with higher income and higher education) was specified as the reference category when present; otherwise, the highest-income and highest-education level of the available group served as reference.^22^ We computed contrasts for each socially disadvantaged group versus the reference, reporting point estimates, 95% CI, and q-values. We calculated Benjamini-Hochberg false discovery rate (FDR) to correct for multiple testing separately across contrasts for pain severity and interference. Using an FDR of 5%, q<0.05 was considered statistically significant.

To evaluate the intersectionality of race, income, education chronic pain, pairwise intercorrelation was quantified using Cramer's V and multicollinearity assessed using variance inflation factors (VIF). The Lindeman-Merenda-Gold (LMG) method was used to evaluate the independent contributions of race and ethnicity, income, and education to explained variance in pain outcomes (*relaimpo* package),^23^ after removing the variance attributable to covariates from regression models. As a linear model is required for LMG, a linear probability model was used for the decomposition of pain interference. Bootstrap 95% CIs were estimated using 1000 permutations.

We performed causal mediation analysis to evaluate potential pathways through which social disadvantages may influence pain outcomes using Mediation R package.^24,25^ Mediation analyses assume sequential ignorability, outcome consistency, and no mediator-outcome confounding.^24,25^ We selected acute and one-year follow-up candidate mediators within the SCIMS dataset that have been linked to traumatic SCI or chronic pain outcomes^7,14,15,26-30^ and organized in a biopsychosocial framework. Biological and rehabilitation-related mediators included: spinal surgery, days in rehabilitation, FIM score at rehabilitation admission and discharge (Functional Independence Measure; an 18-item scale assessing functional ability in daily living), rehospitalizations, days rehospitalized, pressure ulcers, diabetes, and hours in bed. Psychological-related mediators included: depression severity, sleep difficulties, life satisfaction, perceived health status,. Social and structural-related mediators included: health insurance type (acute and one-year), physical independence, CHART mobility score (Craig Handicap Assessment and Reporting Technique; a measure of community participation across mobility, occupation, and social integration domains), days out of house, recreation hours, and alcohol use. Mediators were further classified by assessment timepoint (acute phase or one-year follow-up) as detailed in Supplemental Table 3.

For each mediator, we estimated the average causal mediation effects (ACME), average direct effects (ADE), and proportion mediated using type-appropriate mediator and outcome models (linear for pain severity; logistic for pain interference). We used nonparametric bootstrapping (5000 simulations) with bias-corrected and accelerated 95% CI for estimation. To control for multiple testing, ACME p-values were adjusted within each comparison using the Benjamini–Hochberg FDR. We defined a significant mediator as q<0.05 plus an ∣ACME∣≥0.15 for pain severity (continuous scale) or ∣ACME∣≥0.015 for pain interference (probability/log-odds scale). Reporting of mediation analyses followed AGReMA Mediation Analysis Reporting Guidelines.^31^

## Results

We included 3514 individuals with traumatic SCI with a median age of 43.0 (interquartile range [IQR]: 30) and 79% were male (2773/3514) (Supplemental Figure 3). Inclusion criteria requiring complete sociodemographic and outcome variables resulted in injury dates spanning 2010–2020 for the final cohort. Compared to excluded participants, included participants were more likely to have higher income (73.3% vs. 64.2%; OR 1.53, 95%CI: 1.25–1.88) and higher education (98.0% vs. 96.0%; OR 2.00, 95%CI: 1.54–2.60), and less likely to be non-Hispanic Black (20.1% vs. 24.6%; OR 0.77, 95%CI: 0.70–0.85); sex did not differ (p=0.13). Full details on participants and missing outcomes are included in Supplemental Material.

Sixty-four percent were non-Hispanic White (2262/3514) and 20% (708/3514 were non-Hispanic Black (Table 1). Twenty-seven percent of the cohort was classified as lower income (939/3514) and 63% (2224/ 3514) as having lower education. AIS grade indicated 33% (1146/3514) as A, 12% (424/3514) as B, 16% (561/3514) as C, and 39% (1383/3514) as D. The most common traumatic SCI etiology occurred from vehicular collisions (39%, 1369/3514). Details for characteristics stratified by race, education, and income are included in Supplemental Tables 4-6.

### Pain severity and pain interference were significantly higher among socially disadvantaged groups

**Fig. 1.**
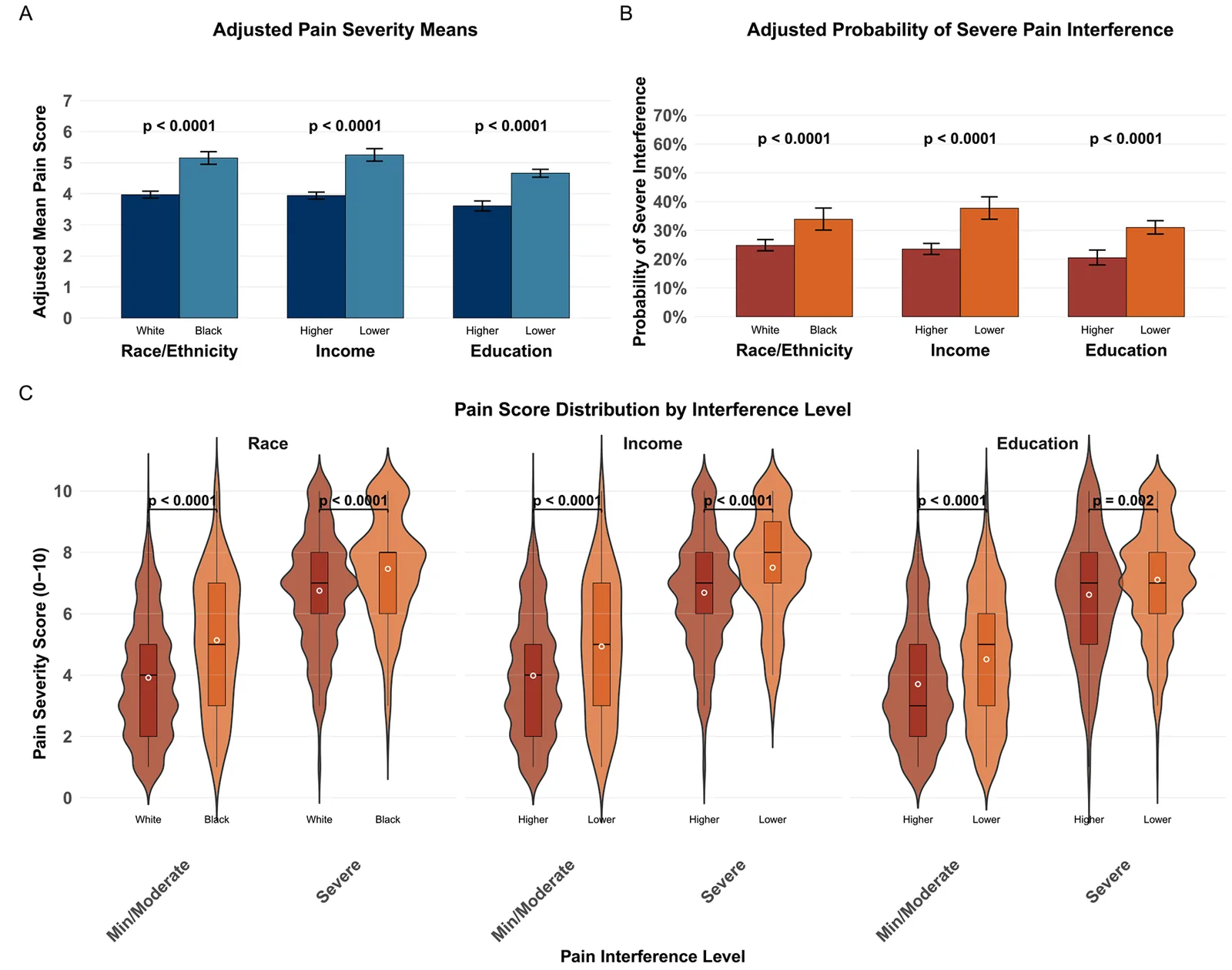
Adjusted Pain Severity and Pain Interference one-year post-traumatic SCI adjusted by race, income, and education. A) Adjusted mean pain severity scores by dicohotomized race, income and education groups with 95% confidence intervals and p-values from pairwise comparisons. B) Adjusted probability of low/moderate and severe pain interference dicohotomized race, income and education groups. C) Violin plots show unadjusted pain score distributions by interference level; white dots indicate means, black boxes show quartiles. Panels A and B control for AIS grade, age, sex, BMI, etiology, and neurological level of injury. Reference groups: Non-Hispanic White, higher income, and higher education.

Non-Hispanic Black participants had higher pain scores than non-Hispanic White participants (estimated marginal mean [M]=5.15, 95%CI: 4.95–5.36 vs. M=3.97, 95%CI: 3.86–4.08 for non-Hispanic White, p<0.0001) (Fig. 1a). Similarly, lower-income participants had higher pain severity compared to higher-income participants (M=5.25, 95%CI: 5.05–5.45 vs. M=3.94, 95%CI: 3.83–4.05, p<0.0001); lower-education participants also had higher pain severity than those with higher education (M=4.66, 95%CI: 4.53–4.79 vs. M=3.61, 95%CI: 3.45–3.77; p<0.0001). Adjusted mean pain severity and pain interference across additional ethnicity and racial groups is included in Supplemental Figure 4. Sensitivity analyses using alternative cut-offs for income and educational levels supported consistent differences in pain outcomes across definitions (Supplemental Figure 5).

Pain interference showed similar differences, with higher rates of severe pain interference among non-Hispanic Black compared to non-Hispanic White (33.8%, 95%CI: 30.1–37.7% vs. 24.8%, 95%CI: 22.9–26.8%, p<0.0001), low-income compared to high-income (37.7%, 95%CI: 33.9–41.6% vs. 23.5%, 95%CI: 21.6–25.5%), and lower-education compared to higher-education (31.0%, 95%CI: 28.7–33.4% vs. 20.4%, 95%CI: 18.0–23.2%; p<0.0001) (Fig 1). Similarly, in supplemental analyses of smaller racial/ethnic subgroups (<10% of cohort), we note that Hispanic Black participants with lower income had 2.20-point higher pain severity (95%CI: 0.824–3.57, q=0.025) compared to non-Hispanic White participants with higher income (additional details included in Supplemental Figures 6-7). Participants with severe pain interference reported significantly higher pain severity scores for race, income, and education (all p<0.001, Fig 1).

### Race, income, and education have compounding effects on pain severity and pain interference

**Fig. 2.**
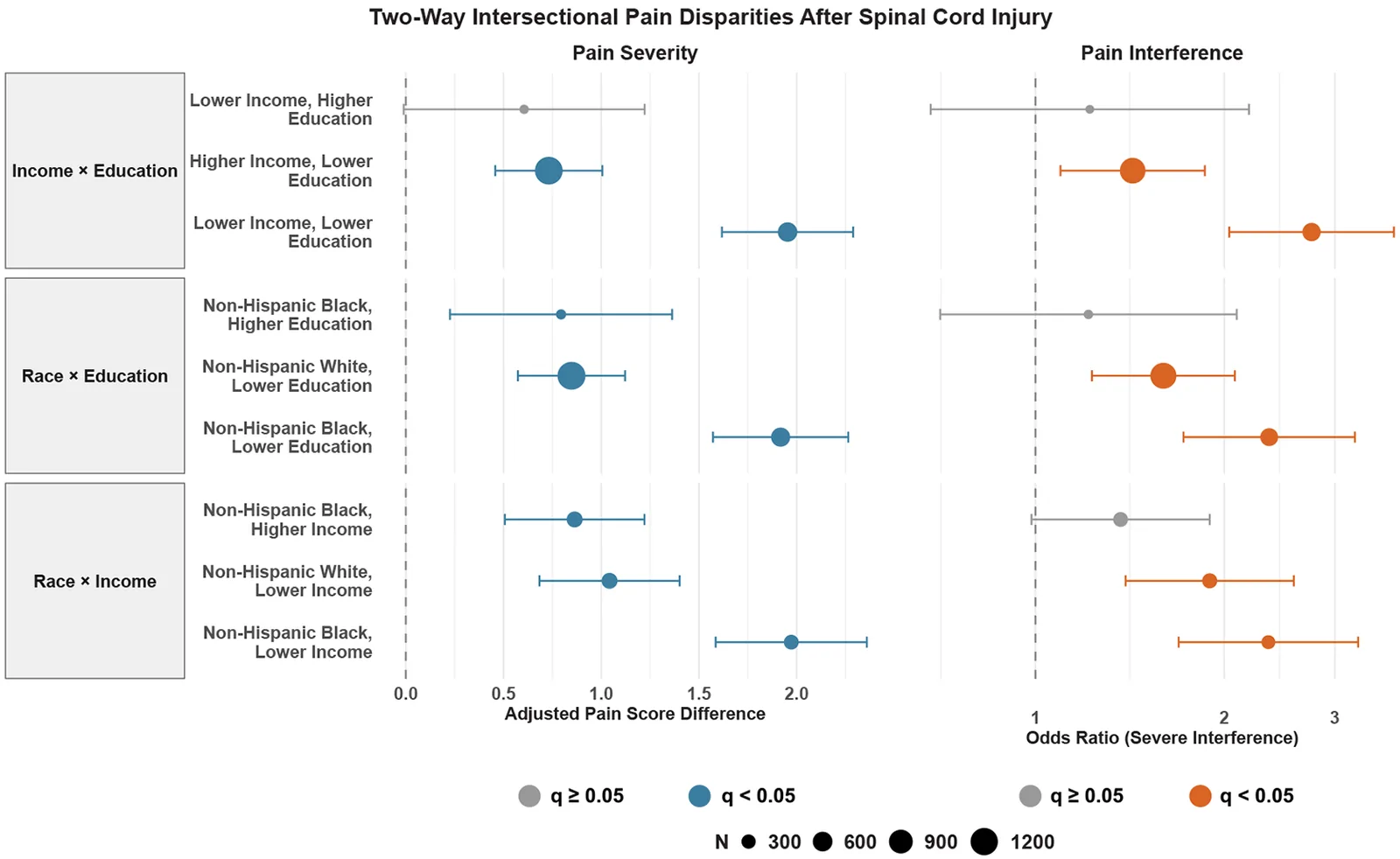
Two-way intersectional analyses of differences in chronic pain severity and pain interference one-year post-traumatic SCI. A) Adjusted mean differences in pain severity scores comparing intersectional groups to corresponding reference groups. B) Odds ratios for severe pain interference comparing intersectional groups to corresponding reference groups. All models control for AIS grade, age at injury, sex, BMI, injury etiology, and neurological injury level. Reference groups: Non-Hispanic White, higher-income (race × income), Non-Hispanic White, higher-education (race × education), higher-income, higher-education (income × education). Point size represents the number of participants in each category. Statistical significance determined using FDR correction applied separately within each intersection type (q < 0.05). Error bars represent 95% confidence intervals.

Non-Hispanic Black participants with lower income had a 1.97-point higher pain severity scores (95%CI: 1.65–2.29, q<0.0001, Fig 2 old higher odds of severe pain interference (95%CI: 1.79–3.10, q<0.0001, Fig 2) compared to non-Hispanic White participants with higher income. Similarly, non-Hispanic Black participants with lower education had a 1.91-point higher pain severity scores (95%CI: 1.63–2.21, q<0.0001) and 2.36-fold higher odds of severe pain interference (95%CI: 1.82–3.06, q<0.0001) compared to non-Hispanic White participants with higher education.

**Fig. 3.**
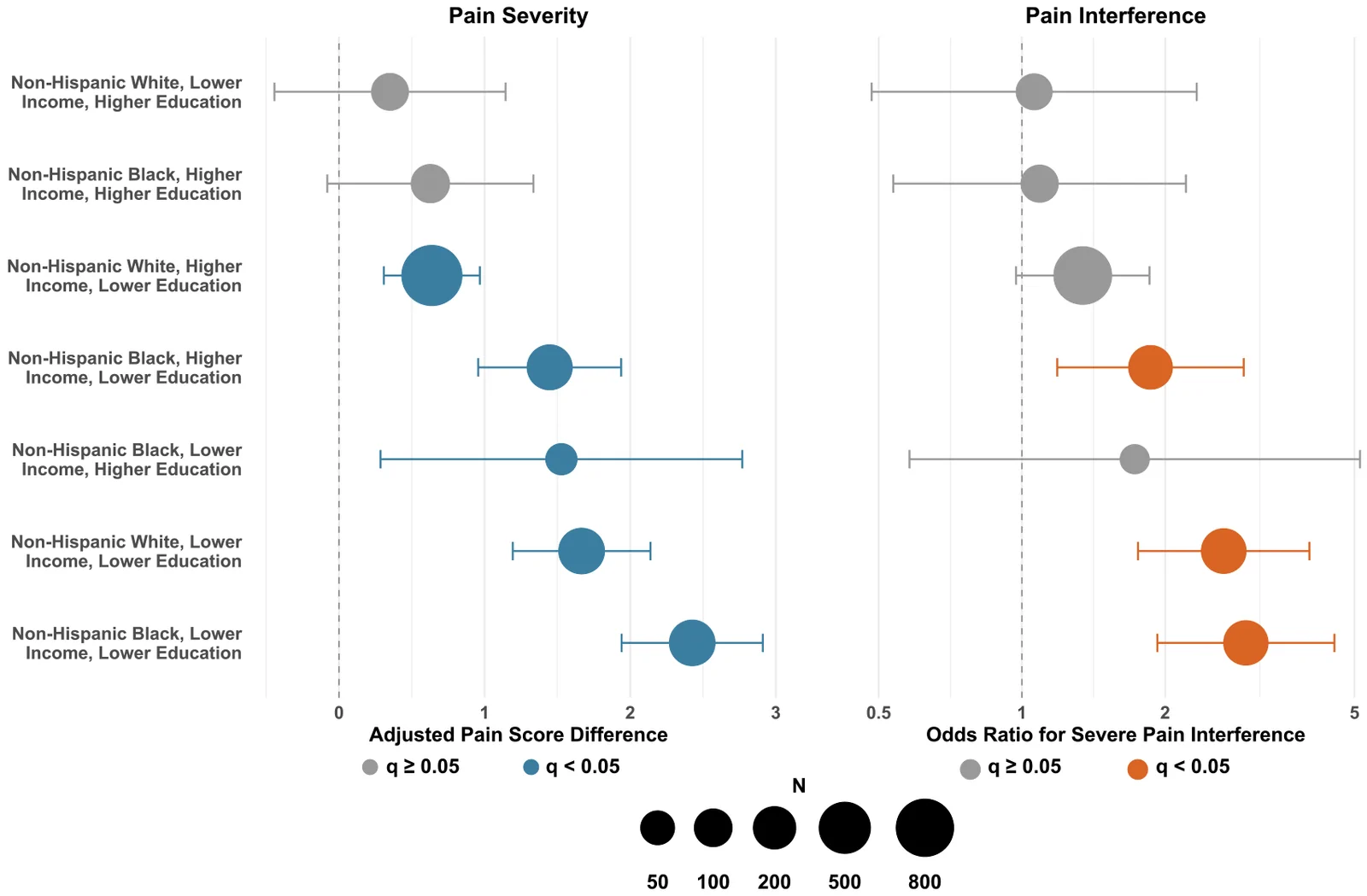
Three-way intersectional analyses of differences in chronic pain severity and pain interference one-year post-traumatic SCI. A) Adjusted mean difference in pain severity scores evaluating race × income × education intersectional groups compared to Non-Hispanic White, higher-income, and higher-education participants. B) Odds ratios for severe pain interference comparing race × income × education intersectional groups compared to Non-Hispanic White, higher-income, and higher-education participants. All models control for AIS grade, age at injury, sex, BMI, injury etiology, and neurological injury level. Point size represents the number of participants in each category. Statistical significance determined using FDR correction applied separately within each intersection type (q < 0.05). Error bars represent 95% confidence intervals.

Three-way intersectional analyses found that non-Hispanic Black participants with lower income and lower education had a 2.43-point higher pain severity scores (95%CI: 2.06–2.79, q<0.0001, Fig 3old higher odds of severe pain interference (95%CI: 2.15–4.07, q<0.0001, Fig 3) compared to non-Hispanic White participants with higher income and higher education. Non-Hispanic White participants with lower education and lower income also had a 1.66-point higher pain severity scores (95%CI: 1.31–2.02, q<0.0001) and 2.66-fold higher odds of severe pain interference (95%CI: 1.95–3.62, q<0.0001) compared to non-Hispanic White participants with higher income and higher education.

Pairwise intercorrelations were low-to-moderate (Cramer's V: Race × Income = 0.291, Race × Education = 0.212, Income × Education = 0.250) with low VIF values (1.14–1.16). Income was the largest independent contributor to pain severity (39.4%) and pain interference (52.0%), followed by education (30.3% and 32.9%, respectively) and race (30.3% and 15.1%, respectively). Full LMG weights and bootstrap 95% CIs are reported in Supplemental Table 7.

### Physical independence, perceived health, and insurance type mediate a portion of differences in chronic pain one-year post-SCI

**Fig. 4.**
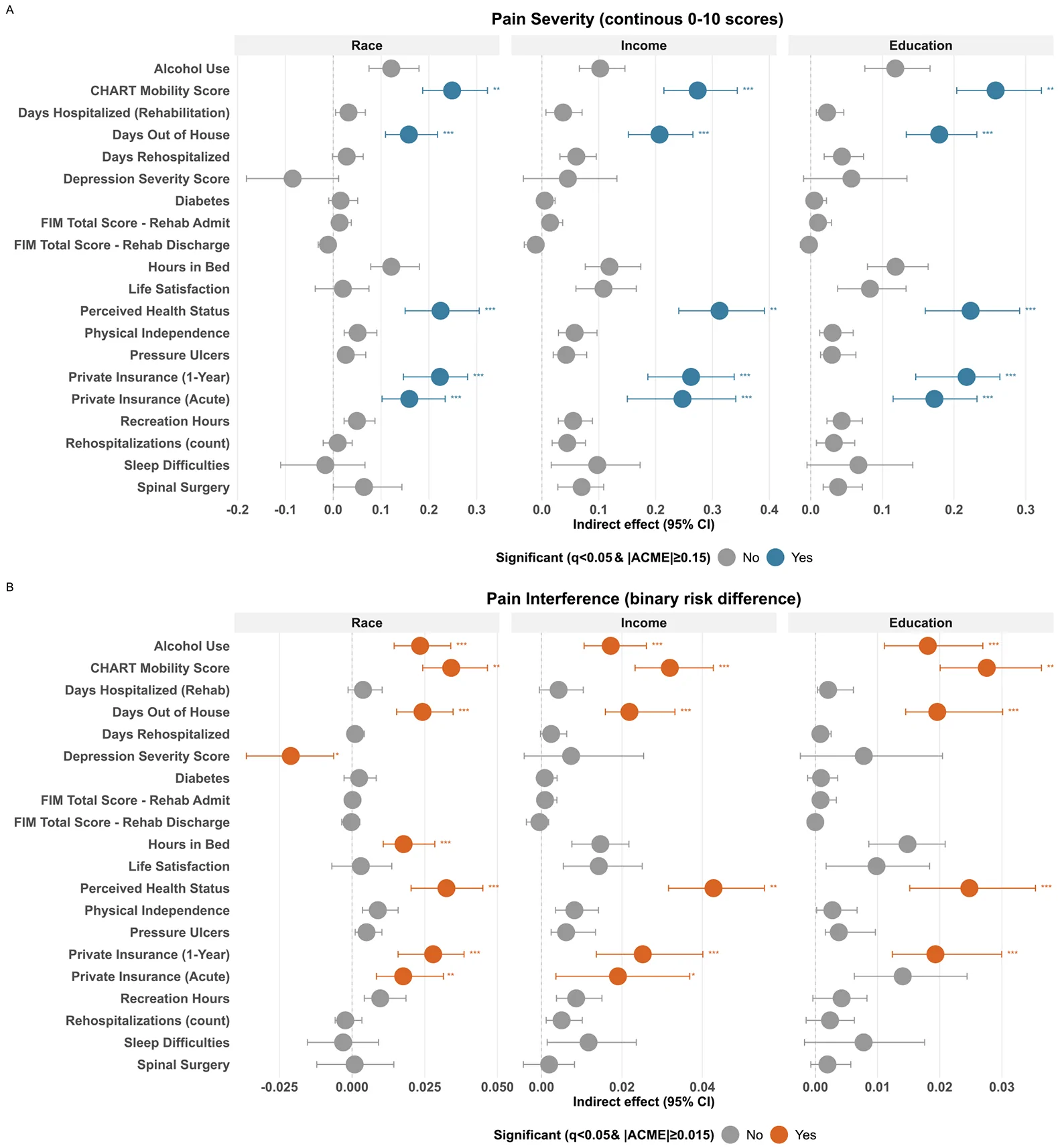
Mediation of differences in chronic pain severity and pain interference one-year post-traumatic SCI. Forest plots show average causal mediation effects (ACME) with 95% bias-corrected and accelerated bootstrap confidence intervals (horizontal bars). Points indicate average causal mediation effect estimates for differences by race (non-Hispanic Black vs non-Hispanic White), income (low vs high), and education (low vs high) for (A) pain severity (0–10 scale, continuous) and (B) severe pain interference (dichotomized, log odds ratios). Individual mediator significance thresholds for pain severity include q < 0.05 and ACME ≥ 0.15, and pain interference thresholds include q < 0.05 and ACME ≥ 0.015. Significant mediators are shown in blue for pain severity and orange for pain interference; nonsignificant in gray. *q<0.05, **q<0.01, ***q<0.001.

Physical mobility (CHART scores) partially mediated pain severity differences related to race, income, and education, accounting for 20.0% of the race difference (ACME=0.249, 95%CI: 0.187–0.323), 22.5% of the education difference (ACME=0.258, 95%CI: 0.204–0.322), and 20.6% of the income difference (ACME=0.274, 95%CI: 0.215–0.344; Fig 4). Perceived health status similarly mediated all three pain severity differences, explaining 18.2% of the race gap (ACME=0.224, 95%CI: 0.150–0.305), 19.3% of the education gap (ACME=0.223, 95%CI: 0.160–0.292), and 23.9% of the income gap (ACME=0.312, 95%CI: 0.241–0.391). Private insurance at one-year post-injury mediated race (17.9%; ACME=0.223, 95%CI: 0.147–0.281), education (18.9%; ACME=0.218, 95%CI: 0.147–0.264), and income (20.3%; ACME=0.262, 95%CI: 0.186–0.338) differences. Days out of house mediated race (12.9%; ACME=0.158, 95%CI: 0.109–0.218), education (15.7%; ACME=0.179, 95%CI: 0.133–0.232), and income (15.7%; ACME=0.207, 95%CI: 0.152–0.266) differences. Private insurance at acute hospitalization additionally mediated all three pain severity differences (13.8–20.1%; ACME range: 0.159–0.247); all q<0.001.

For pain interference, physical mobility again accounted for the largest mediated proportions: 35.2% of the race difference (ACME=0.034, 95%CI: 0.024–0.047), 26.8% of the education difference (ACME=0.028, 95%CI: 0.020–0.036), and 22.2% of the income difference (ACME=0.032, 95%CI: 0.023–0.043; Fig 4). Perceived health status explained 33.6% of the race difference (ACME=0.032, 95%CI: 0.020–0.045), 24.1% of the education difference (ACME=0.025, 95%CI: 0.015–0.035), and 31.1% of the income difference (ACME=0.043, 95% CI: 0.032–0.055). Days out of house, private insurance (one-year), and alcohol use each mediated race, education, and income differences in pain interference (% mediated range: 12.3–25.4%; ACMEs: 0.017–0.028; all q<0.001, except private insurance for income: q=0.03). Hours in bed (18.5%) and depression severity (26.1%) additionally mediated the race difference in pain interference specifically. Additional mediation results are in Supplemental Table 8.

## Discussion

Chronic pain is a disabling consequence of traumatic SCI affecting nearly 70% of all individuals.^1^ In this multicenter retrospective cohort study of 3514 participants across the US, we found significant and intersecting differences in pain severity and pain interference one-year post-traumatic SCI based on race, income, and education. Significant differences in pain outcomes were present, even after controlling for other demographic and injury characteristics. Each social disadvantage, including being non-Hispanic Black, lower income, or less educated was associated with approximately 1-point higher pain severity score and 10% increased odds of severe pain interference compared to counterparts without these social disadvantages. When combined, intersecting social disadvantages demonstrated even greater increases in pain severity and pain interference one-year post-traumatic SCI. Income most strongly explained the variance in pain outcomes (40–52%), though low collinearity across variables indicates race, income, and education each represent distinct dimensions to pain outcomes. Our mediation analyses demonstrated that mobility scores, days out of the house, private health insurance, and perceived health status significantly mediated the associations between race, income, and education and pain outcomes one year following traumatic SCI.

### Intersectional effects of race, income, and education on chronic pain outcomes following traumatic SCI

Prior studies, which often only evaluate single levels of social disadvantage, report clear and consistent differences across outcomes and treatments in individuals from socially disadvantaged backgrounds following traumatic SCI. Such differences include lower rates of inpatient rehabilitation admission, reduced physical independence and social integration, higher rehospitalizations rates, higher treatment costs, higher levels of depression, more days in poor health, more difficulties with mobility, lower quality of life, higher unemployment, and higher mortality.^7,15,32^ While race income, and education each contributed to pain outcomes independently, income explained the most variance, suggesting economic factors may be a particularly important consideration in chronic pain following traumatic SCI.

Factors associated with social disadvantage are complex and may multiplicatively contribute to increased incidence and severity of chronic pain and SCI;^33^ however, literature examining intersecting social disadvantages in both chronic pain and SCI is limited. Recent work in individuals with a broad range of chronic pain conditions identified distinct subgroups characterized by intersecting levels of social disadvantage, with disproportionately higher pain-related disparities associated with minoritized race, disability status, older age, low educational attainment, and unemployment.^34^ In our study, non-Hispanic Black participants with lower income and lower education have an estimated 2.43-point higher pain severity (on a 10-point scale) and nearly three-fold increased odds of severe pain interference one-year after traumatic SCI compared to non-Hispanic White, higher-income, higher-education participants. Although less severe in magnitude, non-Hispanic White participants with lower income and lower education also had higher pain severity and interference compared to their higher income, more educated counterparts (1.66-point higher severity; 2.66-fold higher interference), consistent with compounding effects of socioeconomic disadvantage across racial groups.

### Individual and structural pathways mediating differences in chronic pain outcomes following traumatic SCI

It is important to move beyond the documentation of potential disparities and towards identifying pathways to inform more effective treatment strategies for historically disadvantaged groups.^35,36^ Using causal mediation analyses, we identified possible mediating pathways explaining up to 35% of the differences in chronic pain severity and pain interference one-year following traumatic SCI. Physical mobility, days out of the house, perceived health status, and private health insurance each significantly mediated pain outcomes related to race, income, and education, likely representing shared mechanisms through which different forms of social disadvantage contribute to worse chronic pain outcomes post-SCI.^37^

Disparities in recovery following SCI are well-documented at both the individual and structural levels.^38^ Individuals experiencing disparities in health outcomes following SCI consistently report barriers to transportation, community reintegration, medical equipment access, and higher rates of psychological distress,^14,15,28,39^ which can be directly influenced by structural racism, limited income, and lower health literacy for those with less education.^40,41^ Furthermore, limited access to healthcare resources and inadequate medical insurance coverage have also been associated with worse health outcomes after SCI.^18,32^ The identified mediating pathways further support these findings indicating that equitable pain management requires a comprehensive biopsychosocial approach. The results of our mediation analyses suggest that effective pain management following traumatic SCI need not only include individual clinical treatments, such as medication and physical therapy, but also consider upstream structural challenges, including healthcare access, transportation needs, and community integration to reduce the unequal burden of chronic pain following SCI.

Our mediating factors also highlight potential priorities for clinicians and policymakers for reducing chronic pain disparities following traumatic SCI. Observations across multiple chronic pain conditions suggest that the patients with the *most need* and *most severe* outcomes are *the least likely to be treated* and may be *the most likely to receive inadequate treatment* if they receive any treatment at all.^42^ Interventions addressing individual-level factors, such as increasing access to physical therapy, rehabilitation services, and community reintegration may help alleviate mobility-related disadvantages associated with traumatic SCI-related chronic pain in our cohort.^43^ Interventions targeting perceived health status should also be considered. Patient education and cognitive behavioral interventions in participants with chronic pain post-SCI demonstrated improved pain outcomes and increased activity participation,^44^ representing a relevant treatment approach given that perceived health status mediated differences up to 24% of pain severity and 33% of pain interference in our cohort. In addition to individual-level changes, expanding access to post-injury insurance coverage, transportation access to leave the house, and community reintegration resources at the institutional and policy levels may further reduce the disproportionate burden of chronic pain for historically disadvantaged individuals following SCI. As geographical identifiers are not included in the open access SCIMS dataset, we were unable to account for potential between center differences which may limit generalizability of our findings or the ability to make inferences on the individual subject level.

There is a potential economic benefit for addressing disparities in chronic pain outcomes following traumatic SCI, as the societal costs are substantial. In the US, lifetime costs associated with SCI range from $1.4 – $6.1 million per person, as of 2023.^45^ Individuals with higher levels of SCI-related pain have demonstrated worse functional outcomes and higher health-care resources and costs.^46^ Our findings align with current guidelines that advocate for comprehensive initiatives that integrate both individual-level rehabilitation with systemic policy change.^47^ By addressing targeted mediating factors of SCI outcomes, clinicians and policymakers can implement a more targeted approach for reducing disparities in chronic pain following traumatic SCI.

### Strengths and limitations

Our data include a large, nationally representative sample of participants across the US one-year post-traumatic SCI. There are, however, limitations to consider. While our analyses focused on non-Hispanic White and Black participants due to sample sizes, representation of certain racial groups remained too low to be included in many of our analyses, which highlights a common limitation to further improve external validity.^48^ We have included initial exploration of these identities in our supplemental material, but further study is warranted to understand the relative risk for pain following traumatic SCI in all other racial/ethnic groups. As the SCIMS database does not include direct measures of structural racism, race served as a proxy for structural disadvantage of pain outcomes in this study. Selection bias is possible, as included participants had higher income and education compared to excluded participants. However, because both higher income and higher education are associated with lower pain outcomes, our reported effects for race, income, and education on pain severity and interference are likely to be conservative estimates of population differences.

While pain severity was assessed using a continuous scale, the dichotomized pain interference scores may reduce the granularity of the findings. Pain measures queried pain levels within the past 4 weeks, which aligns with measures of high impact chronic pain^49^ but differs from the differs from the traditional chronic pain definition of ≥3 months. Our mediation analyses identified factors underlying pain differences, but compounding effects and unmeasured factors should be considered (e.g., access to rehabilitation, structural racism, and medication use). More detailed pain phenotyping including pain classification (neuropathic, nociceptive) and localization may add additional important details.

## Conclusions

We identified novel differences in pain severity and pain interference one-year post-traumatic SCI disproportionately affecting individuals with intersecting social disadvantages, including race, income, and education. These differences are partially mediated by modifiable factors, including mobility limitations, perceived health status, and private health insurance. These findings provide increased granularity for chronic pain disparities and highlight the importance of addressing both individual and societal contributions that may influence the unequal burden of chronic pain following traumatic SCI.

## Supplementary Material

MMC1

## Figures and Tables

**Table 1:** Participant characteristics.

Characteristic	Overall (n = 3514)
**Age at Injury (years)**	43.0 (28.0, 58.0)
**Body Mass Index**	26.0 (22.7, 30.1)
**Pain Severity Score (0–10)**	4.0 (2.0, 7.0)
**Days from Injury to Admission**	8.0 (1.0, 17.0)
**Length of Stay—Rehabilitation** ^1^	42.0 (28.0, 62.0)
**Race/Ethnicity**	
Non-Hispanic White	2262 (64%)
Non-Hispanic Black	708 (20%)
**Income Status**	
*Higher Income*	2575 (73%)
*Lower Income*	939 (27%)
**Education Level**	
*Lower Education*	2224 (63%)
*Higher Education*	1290 (37%)
**Sex**	
*Male*	2773 (79%)
*Female*	738 (21%)
*Other/Transgender*	3 (0%)
**AIS Grade**	
A	1146 (33%)
B	424 (12%)
C	561 (16%)
D	1383 (39%)
E	0 (0%)
**Injury Etiology**	
*Vehicular*	1369 (39%)
*Falls*	1189 (34%)
*Violence*	424 (12%)
*Sports & Recreation*	333 (9%)
*Medical/Surgical*	150 (4%)
*Pedestrian*	26 (1%)
*Other*	23 (1%)
**Neurological Level at Discharge**	
*Paraplegia, incomplete*	2080 (59%)
*Paraplegia, complete*	1142 (32%)
*Paraplegia, minimal deficit*	288 (8%)
*Tetraplegia, incomplete*	4 (0%)
**Pain Interference with Work** ^2^	
*Not at all*	596 (20%)
*A little bit*	897 (30%)
*Moderately*	678 (23%)
*Quite a bit*	559 (19%)
*Extremely*	252 (8%)

Data are reported as Median (Q1, Q3) and number of participants (n) (%).

1: missing n = 36

2: missing n = 532.

## Data Availability

The data are available for download from the National Spinal Cord Injury Model Systems Database (https://www.nscisc.uab.edu/Research/NSCISC_DatabasePublicUse).

## Appendix A. Supporting information

Supplementary data associated with this article can be found in the online version at doi:10.1016/j.jpain.2026.106371.

## References

[1] Hunt C, Moman R, Peterson A, Prevalence of chronic pain after spinal cord injury: a systematic review and meta-analysis. Reg Anesth Pain Med. 2021;46(4):328–336. 10.1136/rapm-2020-101960. 33408161

[2] Simpson LA, Eng JJ, Hsieh JTC, Wolfe DL, Spinal Cord Injury Rehabilitation Evidence. Research Team. The health and life priorities of individuals with spinal cord injury: a systematic review. J Neurotrauma. 2012;29(8):1548–1555. 10.1089/neu.2011.2226. PMC3501530 22320160

[3] Mehta S, McIntyre A, Janzen S, Loh E, Teasell R. Systematic review of pharmacologic treatments of pain after spinal cord injury: an update. Arch Phys Med Rehabil. 2016;97(8):1381–1391.e1. 10.1016/j.apmr.2015.12.023. 26797114

[4] Clark JMR, Cao Y, Krause JS. Risk of pain medication misuse after spinal cord injury: the role of substance use, personality, and depression. J Pain. 2017;18(2):166–177. 10.1016/j.jpain.2016.10.011. 27836813

[5] Marmot M. Social determinants of health inequalities. Lancet. 2005;365(9464):1099–1104. 10.1016/S0140-6736(05)71146-6. 15781105

[6] Prego-Domínguez J, Khazaeipour Z, Mallah N, Takkouche B. Socioeconomic status and occurrence of chronic pain: a meta-analysis. Rheumatology. 2020;60(3):1091–1105. 10.1093/rheumatology/keaa758. 33276382

[7] Odonkor CA, Esparza R, Flores LE, Disparities in health care for Black patients in physical medicine and rehabilitation in the United States: a narrative review. PMR. 2021;13(2):180–203. 10.1002/pmrj.12509. 33090686

[8] DiPiro ND, Murday D, Corley EH, Krause JS. Prevalence of chronic health conditions and hospital utilization in adults with spinal cord injury: an analysis of self-report and South Carolina administrative billing data. Spinal Cord. 2019;57(1):33–40. 10.1038/s41393-018-0185-9. 30089894

[9] Jorge A, White MD, Agarwal N. Outcomes in socioeconomically disadvantaged patients with spinal cord injury: a systematic review. J Neurosurg Spine. 2018;29(6):680–686. 10.3171/2018.5.SPINE171242. 30265226

[10] Saunders LL, Krause JS, Peters BA, Reed KS. The relationship of pressure ulcers, race, and socioeconomic conditions after spinal cord injury. J Spinal Cord Med. 2010;33(4):387–395. 10.1080/10790268.2010.11689717. PMC2964027 21061898

[11] Spinal Cord Injury Model System. Published June 2022. Accessed August 5, 2025. 〈https://www.nscisc.uab.edu/Public_Pages/Database〉.

[12] Crenshaw K. Demarginalizing the intersection of race and sex: a Black feminist critique of antidiscrimination doctrine, feminist theory and antiracist politics. U Chic Leg F. 1989;1989(1):139–167.

[13] Mandelbaum J. Advancing health equity by integrating intersectionality into epidemiological research: applications and challenges. J Epidemiol Community Health. 2020;74(9):761–762. 10.1136/jech-2020-213847. 32527861

[14] Brown S, Saunders L, Krause J. Racial disparities in depression and life satisfaction after spinal cord injury: a mediational model. Top Spinal Cord Inj Rehabil. 2012;18(3):232–240. 10.1310/sci1803-232. PMC3479640 23105915

[15] Botticello AL, Boninger M, Charlifue S, To what extent do neighborhood differences mediate racial disparities in participation after spinal cord injury? Arch Phys Med Rehabil. 2016;97(10):1735–1744. 10.1016/j.apmr.2016.04.007. 27155293

[R16] National Spinal Cord Injury Statistical Center; August 5, 2025. 〈https://bpb-us-w2.wpmucdn.com/sites.uab.edu/dist/f/392/files/2025/01/Definition-and-Eligibility-1.pdf〉. Published 2022. Accessed.

[17] Granger CV, Karmarkar AM, Graham JE, The uniform data system for medical rehabilitation: report of patients with traumatic spinal cord injury discharged from rehabilitation programs in 2002-2010. Am J Phys Med Rehabil. 2012;91(4):289–299. 10.1097/PHM.0b013e31824ad2fd. PMC3392040 22407160

[18] Kim H, Cutter GR, George B, Chen Y. Understanding and preventing loss to follow-up: experiences from the spinal cord injury model systems. Top Spinal Cord Inj Rehabil. 2018;24(2):97–109. 10.1310/sci2402-97. PMC5915110 29706754

[19] Ware JE, Kosinski M, Keller SD. A 12-item short-form health survey: construction of scales and preliminary tests of reliability and validity. Med Care. 1996;34(3):220–233. 10.1097/00005650-199603000-00003 8628042

[20] Von Korff M, DeBar LL, Krebs EE, Graded chronic pain scale revised: mild, bothersome, and high-impact chronic pain. Pain. 2020;161(3):651–661. 10.1097/j.pain.0000000000001758. PMC7097879 31764390

[R21] US Census Bureau; 2025. 〈https://www.census.gov/about/our-research/race-ethnicity/standards-updates.html〉. Published March 28, 2024. Accessed August 20.

[22] Keppel K, Pamuk E, Lynch J, Methodological issues in measuring health disparities, 2 Vital Health Stat. 2005;141:1–16. PMC3681823 16032956

[23] Groemping U. Relative importance for linear regression in R: the package relaimpo. J Stat Softw. 2006;17(1):1–27. 10.18637/jss.v017.i01.

[24] Imai K, Keele L, Tingley D. A general approach to causal mediation analysis. Psychol Methods. 2010;15(4):309–334. 10.1037/a0020761. 20954780

[25] Tingley D, Yamamoto T, Hirose K, Keele L, Imai K. mediation: R package for causal mediation analysis. J Stat Softw. 2014;59(5):1–38. 10.18637/jss.v059.i05.

[26] Escalon MX, Houtrow A, Skelton F, Verduzco-Gutierrez M. Health care disparities add insult to spinal cord injury. Neurol Clin Pract. 2021;11(6):e893–e895. 10.1212/CPJ.0000000000001095. PMC8723932 34992973

[27] Fyffe DC, Deutsch A, Botticello AL, Kirshblum S, Ottenbacher KJ. Racial and ethnic disparities in functioning at discharge and follow-up among patients with motor complete spinal cord injury. Arch Phys Med Rehabil. 2014;95(11):2140–2151. 10.1016/j.apmr.2014.07.398. PMC4374601 25093999

[28] Gary KW, Nicholls E, Shamburger A, Stevens LF, Arango-Lasprilla JC. Do racial and ethnic minority patients fare worse after SCI?: a critical review of the literature. NeuroRehabilitation. 2011;29(3):275–293. 10.3233/nre-2011-0704. 22142762

[29] Kabangu J-LK, Heskett CA, De Stefano FA, Race and socioeconomic disparities persist in treatment and outcomes of patients with cervical spinal cord injuries: an analysis of the national inpatient sample from 2016-2020. World Neurosurg X. 2024; 23, 100384. 10.1016/j.wnsx.2024.100384. PMC11078697 38725975

[30] Krause JS, Saladin LK, Adkins RH. Disparities in subjective well-being, participation, and health after spinal cord injury: a 6-year longitudinal study. NeuroRehabilitation. 2009;24(1):47–56. 10.3233/nre-2009-0453. 19208957

[31] Lee H, Cashin AG, Lamb SE, A guideline for reporting mediation analyses of randomized trials and observational studies: the AGReMA statement. JAMA. 2021;326(11):1045–1056. 10.1001/jama.2021.14075. PMC8974292 34546296

[32] Shakil H, Essa A, Malhotra AK, Insurance-related disparities in withdrawal of life support and mortality after spinal cord injury. JAMA Surg. 2024;159(10):1196–1204. 10.1001/jamasurg.2024.2967. PMC11325240 39141362

[33] Macgregor C, Walumbe J, Tulle E, Seenan C, Blane DN. Intersectionality as a theoretical framework for researching health inequities in chronic pain. Br J Pain. 2023;17(5):479–490. 10.1177/20494637231188583. PMC10722103 38107758

[34] Newman AK, Thorn BE. Intersectional identity approach to chronic pain disparities using latent class analysis. Pain. 2022;163(4):e547–e556. 10.1097/j.pain.0000000000002407. 34252906

[35] Booker SQ, Baker TA, Esiaka D, A historical review of pain disparities research: advancing toward health equity and empowerment. Nurs Outlook. 2023;71(3), 101965. 10.1016/j.outlook.2023.101965. PMC11198876 37023670

[36] Bhattacharya J. Advancing NIH's mission through a unified strategy. National Institutes of Health. Published August 15, 2025. Accessed September 12, 2025. 〈https://www.nih.gov/about-nih/nih-director/statements/advancing-nihs-mission-through-unified-strategy〉.

[37] Widerström-Noga E, Anderson KD, Perez S, Living with chronic pain after spinal cord injury: a mixed-methods study. Arch Phys Med Rehabil. 2017;98(5):856–865. 10.1016/j.apmr.2016.10.018. 27894730

[38] Fyffe D, Botticello A, Myaskovsky L. Vulnerable groups living with spinal cord injury. Top Spinal Cord Inj Rehabil. 2011;17(2):1–9. 10.1310/sci1702-01. PMC3746335 23966760

[39] Celeste-Villalvir A, Kovic C, Argüelles F. The intersectional impact of disability and immigration on health: a health needs assessment of immigrants living with spinal cord injury in Houston, Texas. Community Health Equity Res Policy. 2024;44(2):209–218. 10.1177/2752535x221132445. 36670517

[40] Khazanchi R, Evans CT, Marcelin JR. Racism, not race, drives inequity across the COVID-19 continuum. JAMA Netw Open. 2020;3(9), e2019933. 10.1001/jamanetworkopen.2020.19933. 32975568

[41] Braveman P, Parker Dominguez T. Abandon "race." Focus on racism. Front Public Health. 2021;9, 689462. 10.3389/fpubh.2021.689462. PMC8452910 34557466

[42] Nguyen LH, Dawson JE, Brooks M, Khan JS, Telusca N. Disparities in pain management. Anesthesiol Clin. 2023;41(2):471–488. 10.1016/j.anclin.2023.03.008. 37245951

[43] Gómara-Toldrà N, Sliwinski M, Dijkers MP, Physical therapy after spinal cord injury: a systematic review of treatments focused on participation. J Spinal Cord Med. 2014;37(4):371–379. 10.1179/2045772314Y.0000000194. PMC4116720 24621042

[44] Heutink M, Post MWM, Bongers-Janssen HMH, The CONECSI trial: results of a randomized controlled trial of a multidisciplinary cognitive behavioral program for coping with chronic neuropathic pain after spinal cord injury. Pain. 2012;153(1):120–128. 10.1016/j.pain.2011.09.029. 22100355

[45] Cao Y, Chen Y, DeVivo M. Lifetime direct costs after spinal cord injury. Top Spinal Cord Inj Rehabil. 2011;16(4):10–16. 10.1310/sci1604-10.

[46] Mann R, Schaefer C, Sadosky A, Burden of spinal cord injury-related neuropathic pain in the United States: retrospective chart review and cross-sectional survey. Spinal Cord. 2013;51(7):564–570. 10.1038/sc.2013.34. 23588572

[47] Gkiouleka A, Wong G, Sowden S, Reducing health inequalities through general practice. Lancet Public Health. 2023;8(6):e463–e472. 10.1016/S2468-2667(23)00093-2. 37244675

[48] Dildine TC, Janevic MR, Hood AM, The "External Validity Crisis": now is the time to address the attack on diversity, equity, and inclusion in pain science (Published online) Pain. 2025. 10.1097/j.pain.0000000000003788. 40893021

[49] Herman PM, Broten N, Lavelle TA, Sorbero ME, Coulter ID. Health care costs and opioid use associated with high-impact chronic spinal pain in the United States. Spine. 2019;44(16):1154–1161. 10.1097/BRS.0000000000003033 PMC6758926 31373999

