# Supplementary material for: Applying an intersectional framework to characterize chronic pain disparities one year following traumatic spinal cord injury
Source: J Pain. Author manuscript; Available in PMC 2026 Sep 16. (PMC13575930; doi:10.1016/j.jpain.2026.106371)
Supplement: Supplementary file 1 [file NIHMS2194364-supplement-MMC1.pdf]

Supplemental Material

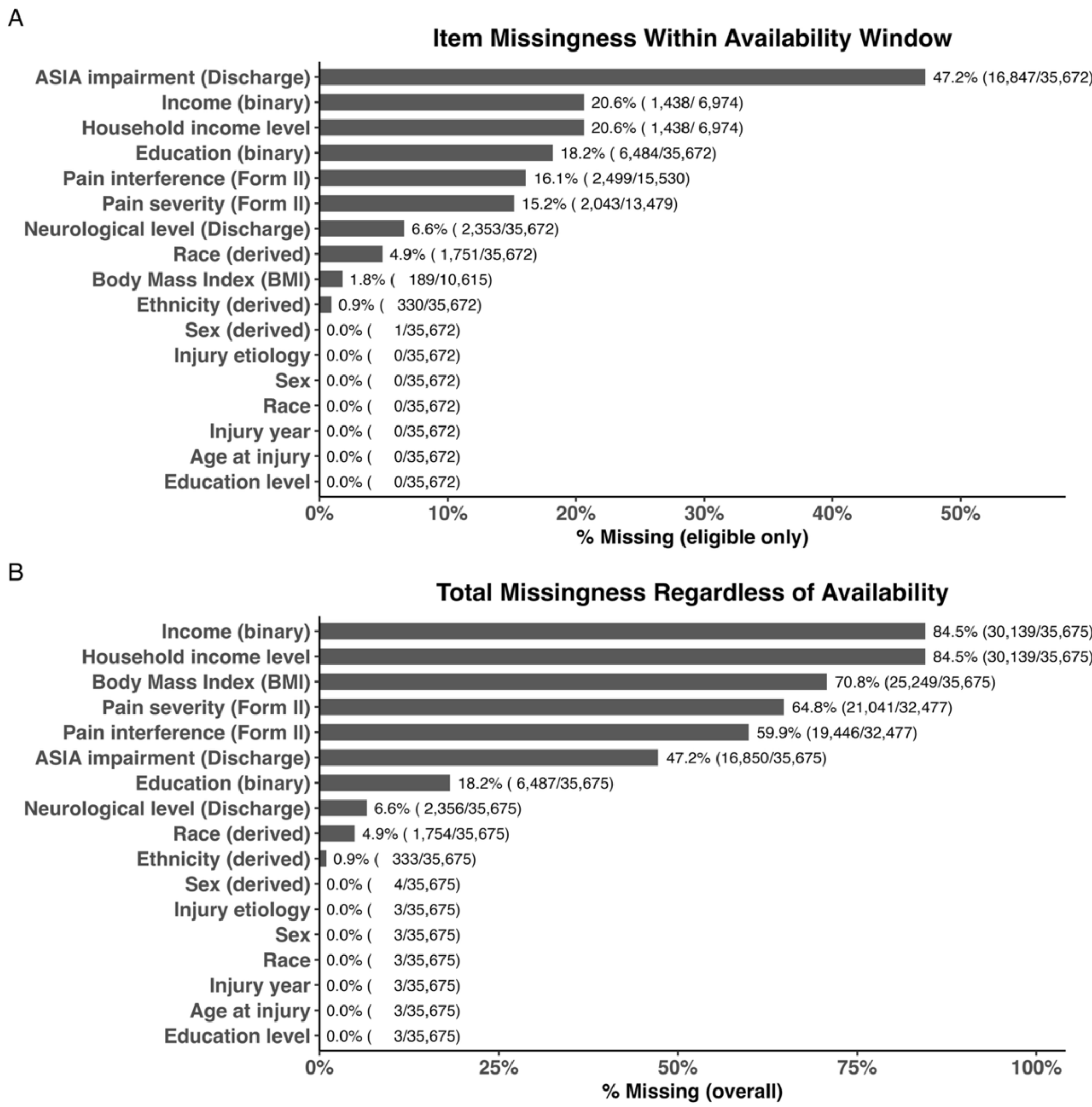

Supplemental Figure 1. Patterns of variable missingness in the SCIMS baseline and one-year datasets. A) shows the proportion of item-level missingness on availability of main variables. Bars indicate the percentage of cases with item nonresponse. B) shows total item missingness regardless of temporal addition into the SCIMS dataset.

(A) Item Missingness by Demographic Group

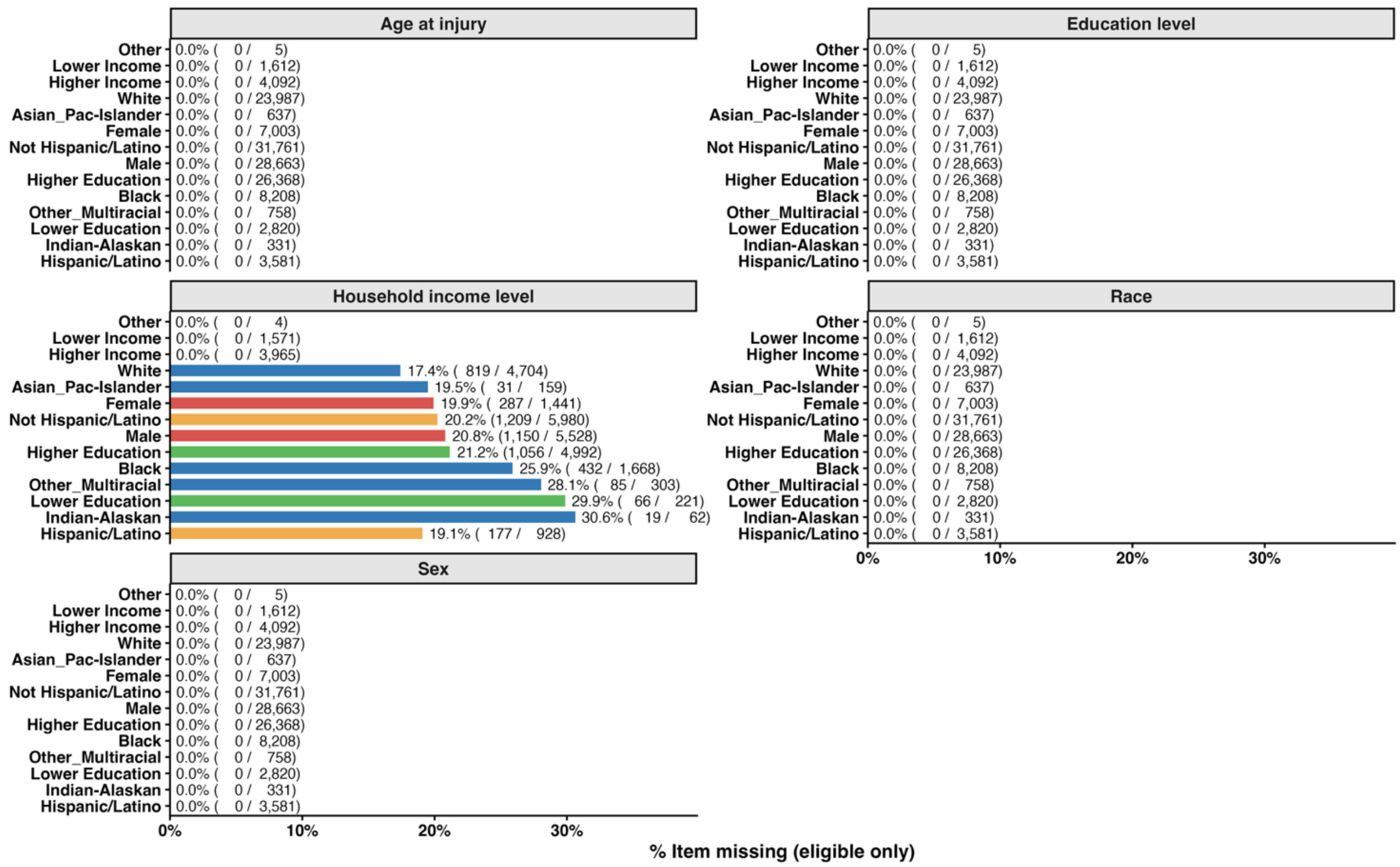

(B) Item Missingness by Demographic Group

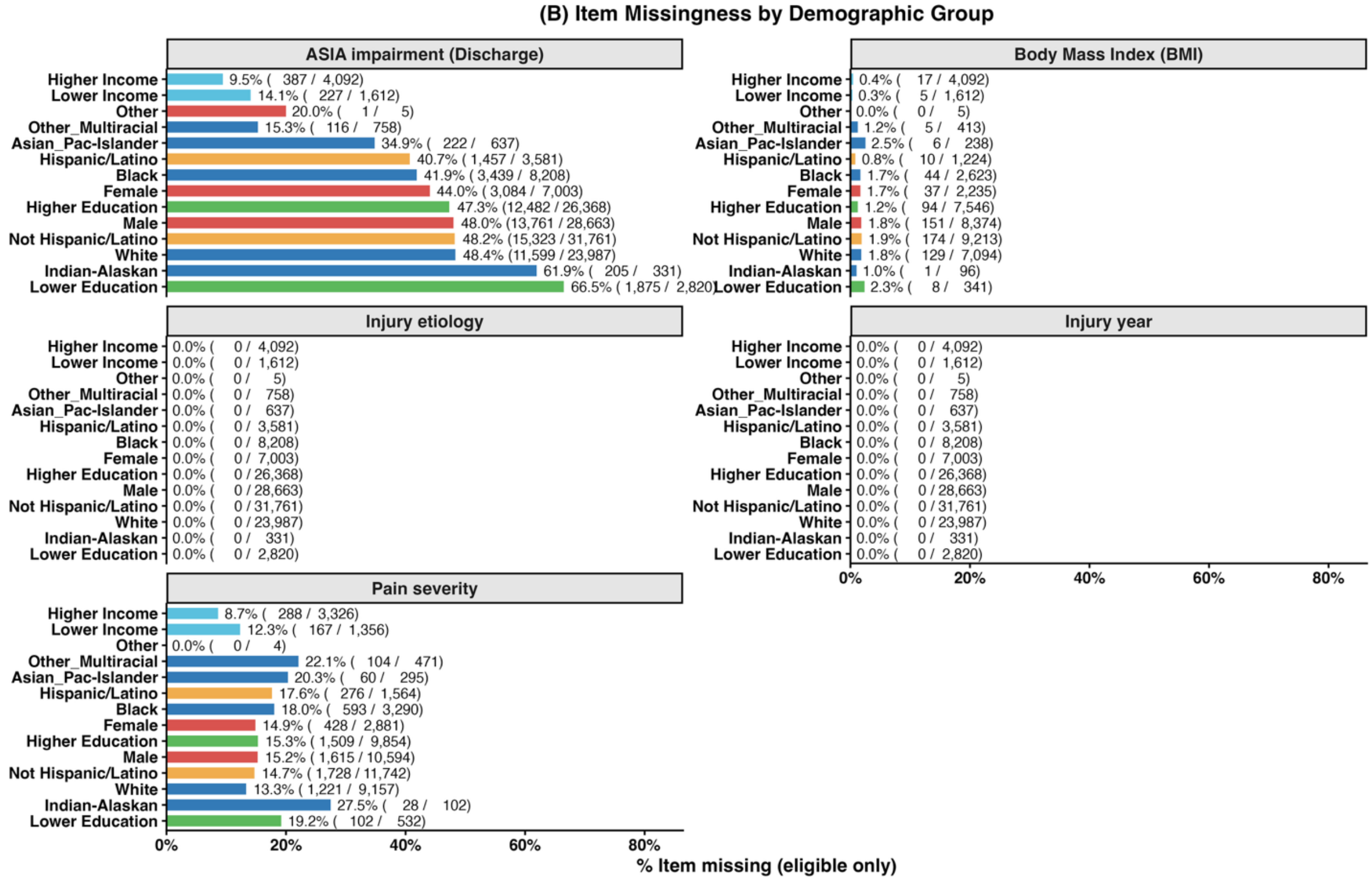

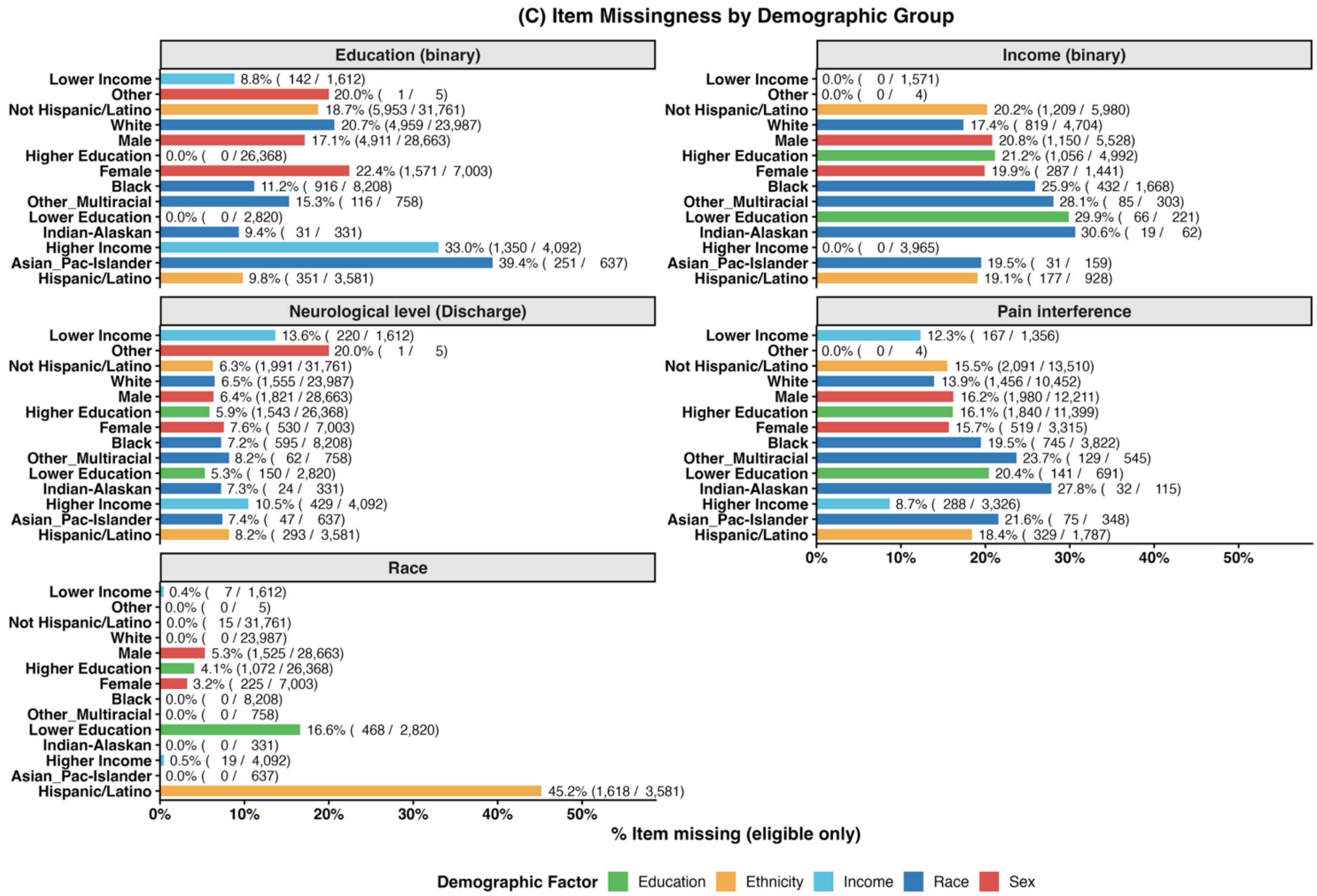

Supplemental Figure 2. Item missingness by demographic groups across primary study variables. The percentage of item missingness among eligible observations within the SCIMS dataset (i.e., records for which the variable was collected at the relevant time point). Within each item, bars represent demographic subgroups with corresponding color coding.

SUPPLEMENTARY RESUTLS

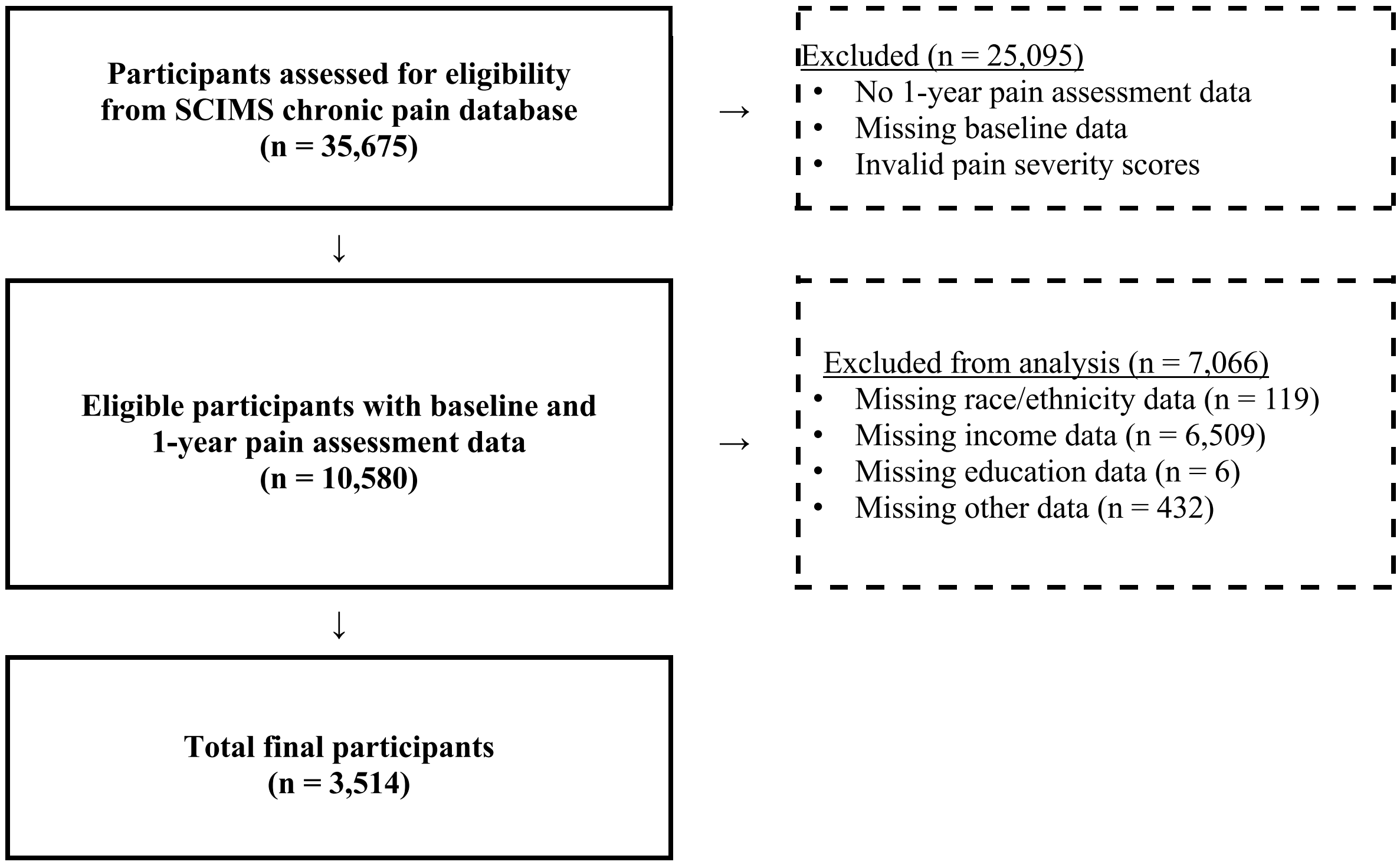

Supplemental Figure 3: CONSORT flow diagram showing participant selection for analysis of racial disparities in chronic pain outcomes from the SCIMS database.

Participant characteristics between included and excluded SCIMS participants:
For all participants eligible for our study within the SCIMS database, included participants were more likely to have higher income (73.3% vs 64.2%; OR 1.53, 95% CI 1.25–1.88) and higher education (98.0% vs 96.0%; OR 2.00, 95% CI 1.54–2.60). There were moderate racial/ethnic differences (p <0.0001) with fewer non-Hispanic Black traumatic SCI participants included in final analysis (20.1% vs 24.6%; OR 0.77, 0.70–0.85). The two samples did not different by sex (p=0.13).
In the secondary analysis restricted to those SCIMS database participants who met the study selection criteria, we identified similar patterns demographic patterns of loss to following up with greater number of non-Hispanic participants, as well as differences income and education levels.

Supplemental Table 1. Analysis of participant selection (complete sample)

Variable	Level	Included	Excluded	OR for inclusion (95% CI)	Omnibus p-value
education	Higher Education	3,442 (98.0%)	6,429 (96.0%)	2.00 (1.54, 2.60)	<0.0001
education	Lower Education	72 (2.0%)	269 (4.0%)	0.50 (0.38, 0.65)	
income	Higher Income	2,575 (73.3%)	294 (64.2%)	1.53 (1.25, 1.88)	0.0001
income	Lower Income	939 (26.7%)	164 (35.8%)	0.65 (0.53, 0.80)	
race_ethnicity	Hisp_Asian_Pac-Islander	1 (0.0%)	6 (0.1%)	0.33 (0.04, 2.71)	<0.0001
race_ethnicity	Hisp_Black	28 (0.8%)	61 (0.9%)	0.90 (0.57, 1.41)	
race_ethnicity	Hisp_Indian-Alaskan	8 (0.2%)	16 (0.2%)	0.98 (0.42, 2.29)	
race_ethnicity	Hisp_Other_Multiracial	83 (2.4%)	163 (2.4%)	1.00 (0.76, 1.30)	
race_ethnicity	Hisp_White	302 (8.6%)	458 (6.7%)	1.32 (1.13, 1.53)	
race_ethnicity	NonHisp_Asian_Pac-Islander	70 (2.0%)	138 (2.0%)	0.99 (0.74, 1.33)	
race_ethnicity	NonHisp_Black	708 (20.1%)	1,696 (24.6%)	0.77 (0.70, 0.85)	
race_ethnicity	NonHisp_Indian-Alaskan	13 (0.4%)	28 (0.4%)	0.91 (0.47, 1.76)	
race_ethnicity	NonHisp_Other_Multiracial	39 (1.1%)	49 (0.7%)	1.57 (1.03, 2.39)	
race_ethnicity	NonHisp_White	2,262 (64.4%)	4,267 (62.0%)	1.11 (1.02, 1.20)	
sex	Female	738 (21.0%)	1,534 (21.7%)	0.96 (0.87, 1.06)	0.1328
sex	Male	2,773 (78.9%)	5,531 (78.3%)	1.04 (0.94, 1.15)	
sex	Other	3 (0.1%)	1 (0.0%)	6.04 (0.63, 58.06)	

Supplemental Table 2. Analysis of participant selection (reduced sample based on eligibility criteria)

Variable	Level	Included	Excluded	OR for inclusion (95% CI)	Omnibus p-value
education	Higher Education	3,442 (98.0%)	412 (95.6%)	2.20 (1.32, 3.69)	0.004
education	Lower Education	72 (2.0%)	19 (4.4%)	0.45 (0.27, 0.76)	
income	Higher Income	2,575 (73.3%)	276 (64.0%)	1.54 (1.25, 1.90)	0.0001
income	Lower Income	939 (26.7%)	155 (36.0%)	0.65 (0.53, 0.80)	
race_ethnicity	Hisp_Asian_Pac-Islander	1 (0.0%)	0 (0.0%)	0.37 (0.01, 9.06)	<0.0001
race_ethnicity	Hisp_Black	28 (0.8%)	7 (1.6%)	0.49 (0.21, 1.12)	
race_ethnicity	Hisp_Indian-Alaskan	8 (0.2%)	1 (0.2%)	0.98 (0.12, 7.86)	
race_ethnicity	Hisp_Other_Multiracial	83 (2.4%)	11 (2.6%)	0.92 (0.49, 1.75)	
race_ethnicity	Hisp_White	302 (8.6%)	88 (20.4%)	0.37 (0.28, 0.48)	
race_ethnicity	NonHisp_Asian_Pac-Islander	70 (2.0%)	13 (3.0%)	0.65 (0.36, 1.19)	
race_ethnicity	NonHisp_Black	708 (20.1%)	97 (22.5%)	0.87 (0.68, 1.11)	
race_ethnicity	NonHisp_Indian-Alaskan	13 (0.4%)	3 (0.7%)	0.53 (0.15, 1.87)	
race_ethnicity	NonHisp_Other_Multiracial	39 (1.1%)	7 (1.6%)	0.68 (0.30, 1.53)	
race_ethnicity	NonHisp_White	2,262 (64.4%)	204 (47.3%)	2.01 (1.64, 2.46)	
sex	Female	738 (21.0%)	88 (20.4%)	1.04 (0.81, 1.33)	0.861
sex	Male	2,773 (78.9%)	343 (79.6%)	0.96 (0.75, 1.23)	
sex	Other	3 (0.1%)	0 (0.0%)	0.86 (0.04, 16.68)	

Candidate Mediator Selection

Supplemental Table 3. Mediator names with timing

Timepoint	SCIMS Variable	Label in Analysis
Acute	AHDaSyRb	Days Hospitalized (Rehabilitation)
Acute	ASpinSrg	Spinal Surgery
Acute	APrimPay	Health Insurance Type
Acute	AFScorRb	FIM Total Score - Rehab Admit
Acute	AFScorDs	FIM Total Score - Rehab Discharge
1-Year Follow-up	BPrUlcer	Pressure Ulcers
1-Year Follow-up	BDiabete	Diabetes
1-Year Follow-up	BRhspNbr	Rehospitalizations (count)
1-Year Follow-up	BrhspDaT	Days Rehospitalized
1-Year Follow-up	BLifSatT	Life Satisfaction
1-Year Follow-up	BAlcRate	Alcohol Use
1-Year Follow-up	BSPHthSt	Perceived Health Status
1-Year Follow-up	BCHHrBed	Hours in Bed
1-Year Follow-up	BCHDaHse	Days Out of House
1-Year Follow-up	BCHHrRec	Recreation Hours
1-Year Follow-up	BCHPITot	Physical Independence
1-Year Follow-up	BCHMbTot	CHART Mobility Score
1-Year Follow-up	BBPHQSDS	Depression Severity Score
1-Year Follow-up	BSleep	Sleep Difficulties
1-Year Follow-up	BPrimPay	Health Insurance Type

Supplemental Table 4: Participant characteristics by race/ethnicity

Characteristic	Non-Hispanic White<br>N = 2,262[1]	Non-Hispanic Black<br>N = 708[1]	p-value[2]
**Age at Injury (years)**	48.0 (30.0, 61.0)	37.0 (25.0, 52.0)	<0.001
**Body Mass Index (kg/m²)**	26.2 (23.0, 30.1)	25.4 (21.9, 30.0)	<0.001
**Pain Severity Score (0-10)**	4.0 (2.0, 6.0)	6.0 (3.0, 7.0)	<0.001
**Days from Injury to Admission**	9.0 (1.0, 17.0)	5.0 (1.0, 16.5)	<0.001
**Injury to Rehab Admit Days**	13.0 (9.0, 23.0)	14.0 (9.0, 26.5)	0.003
**Length of Stay—Rehab**	44.0 (29.0, 64.0)	38.0 (26.0, 56.0)	<0.001
Missing	23	7	
**Income Status**			<0.001
Higher Income	1,881 (83%)	383 (54%)	
Lower Income	381 (17%)	325 (46%)	
**Education Level**			<0.001
Lower Education	1,256 (56%)	565 (80%)	
Higher Education	1,006 (44%)	143 (20%)	
**Sex**			0.052
Male	1,751 (77%)	578 (82%)	
Female	510 (23%)	130 (18%)	
Other/Transgender	1 (0%)	0 (0%)	
Missing	0 (0%)	0 (0%)	
**AIS Grading**			0.002
A	671 (30%)	256 (36%)	
B	256 (11%)	90 (13%)	
C	387 (17%)	95 (13%)	
D	948 (42%)	267 (38%)	
E	0 (0%)	0 (0%)	
**Injury Etiology**			<0.001
Vehicular	918 (41%)	241 (34%)	
Falls	862 (38%)	164 (23%)	
Violence	69 (3%)	261 (37%)	
Sports & Recreation	265 (12%)	15 (2%)	
Medical/Surgical	114 (5%)	22 (3%)	
Other	19 (1%)	2 (0%)	
Pedestrian	15 (1%)	3 (0%)	
**Neurological Level at Discharge**			0.102
Paraplegia, incomplete	1,402 (62%)	402 (57%)	
Paraplegia, complete	682 (30%)	242 (34%)	
Paraplegia, minimal deficit	176 (8%)	63 (9%)	
Tetraplegia, incomplete	2 (0%)	1 (0%)	
**Pain Interference with Work**			0.005
A little bit	584 (26%)	179 (25%)	
Moderately	459 (20%)	124 (18%)	
Not at all	395 (17%)	105 (15%)	
Quite a bit	345 (15%)	132 (19%)	
Not applicable (no pain)	319 (14%)	94 (13%)	
Extremely	138 (6%)	69 (10%)	
Unknown	13 (1%)	5 (1%)	
Declined	6 (0%)	0 (0%)	
Doesn't know	3 (0%)	0 (0%)	

[1]Median (Q1, Q3); n (%)

[2]Wilcoxon rank sum test; Pearson's Chi-squared test

Supplemental Table 5: Participant characteristics by income levels

Characteristic	Higher Income N = 2,575[1]	Lower Income N = 939[1]	p-value[2]
**Age at Injury (years)**	46.0 (30.0, 59.0)	34.0 (24.0, 51.0)	<0.001
**Body Mass Index (kg/m²)**	26.3 (23.1, 30.3)	25.0 (21.8, 29.4)	<0.001
**Pain Severity Score (0-10)**	4.0 (2.0, 6.0)	6.0 (3.0, 7.0)	<0.001
**Days from Injury to Admission**	9.0 (1.0, 17.0)	6.0 (1.0, 18.0)	0.006
**Injury to Rehab Admit Days**	13.0 (9.0, 23.0)	16.0 (9.0, 29.0)	<0.001
**Length of Stay—Rehab**	44.0 (30.0, 64.0)	37.0 (24.0, 56.0)	<0.001
Missing	28	8	
**Education Level**			<0.001
Lower Education	1,427 (55%)	797 (85%)	
Higher Education	1,148 (45%)	142 (15%)	
**Sex**			0.041
Male	2,059 (80%)	714 (76%)	
Female	514 (20%)	224 (24%)	
Other/Transgender	2 (0%)	1 (0%)	
Missing	0 (0%)	0 (0%)	
**AIS Grade**			<0.001
A	773 (30%)	373 (40%)	
B	320 (12%)	104 (11%)	
C	434 (17%)	127 (14%)	
D	1,048 (41%)	335 (36%)	
E	0 (0%)	0 (0%)	
**Injury Etiology**			<0.001
Vehicular	1,040 (40%)	329 (35%)	
Falls	933 (36%)	256 (27%)	
Violence	168 (7%)	256 (27%)	
Sports & Recreation	278 (11%)	55 (6%)	
Medical/Surgical	118 (5%)	32 (3%)	
Pedestrian	17 (1%)	9 (1%)	
Other	21 (1%)	2 (0%)	
**Neurological Level at Discharge**			<0.001
Paraplegia, incomplete	1,584 (62%)	496 (53%)	
Paraplegia, complete	789 (31%)	353 (38%)	
Paraplegia, minimal deficit	199 (8%)	89 (9%)	
Tetraplegia, incomplete	3 (0%)	1 (0%)	
**Pain Interference with Work**			<0.001
A little bit	686 (27%)	211 (22%)	
Moderately	509 (20%)	169 (18%)	
Not at all	452 (18%)	144 (15%)	
Quite a bit	376 (15%)	183 (19%)	
Not applicable (no pain)	385 (15%)	112 (12%)	
Extremely	141 (5%)	111 (12%)	
Unknown	15 (1%)	6 (1%)	
Declined	6 (0%)	3 (0%)	
Doesn't know	5 (0%)	0 (0%)	

[1]Median (Q1, Q3); n (%)
[2]Wilcoxon rank sum test; Pearson's Chi-squared test

Supplemental Table 6: Participant characteristics by education levels

Characteristic	Higher Education N = 1,290[1]	Lower Education N = 2,224[1]	p-value[2]
**Age at Injury (years)**	50.0 (35.0, 63.0)	38.0 (25.0, 54.0)	<0.001
**Body Mass Index (kg/m²)**	26.3 (23.4, 30.2)	25.7 (22.4, 30.0)	<0.001
**Pain Severity Score (0-10)**	3.0 (2.0, 5.0)	5.0 (2.5, 7.0)	<0.001
**Days from Injury to Admission**	9.0 (1.0, 17.0)	8.0 (1.0, 17.0)	0.190
**Injury to Rehab Admit Days**	13.0 (8.0, 22.0)	14.0 (9.0, 26.0)	<0.001
**Length of Stay—Rehab**	44.0 (29.0, 64.0)	42.0 (28.0, 59.0)	<0.001
Missing	17	19	
**Income Status**			<0.001
Higher Income	1,148 (89%)	1,427 (64%)	
Lower Income	142 (11%)	797 (36%)	
**Sex**			<0.001
Male	942 (73%)	1,831 (82%)	
Female	347 (27%)	391 (18%)	
Other/Transgender	1 (0%)	2 (0%)	
Missing	0 (0%)	0 (0%)	
**AIS Grade**			<0.001
A	336 (26%)	810 (36%)	
B	151 (12%)	273 (12%)	
C	214 (17%)	347 (16%)	
D	589 (46%)	794 (36%)	
E	0 (0%)	0 (0%)	
**Injury Etiology**			<0.001
Vehicular	497 (39%)	872 (39%)	
Falls	490 (38%)	699 (31%)	
Violence	47 (4%)	377 (17%)	
Sports & Recreation	170 (13%)	163 (7%)	
Medical/Surgical	64 (5%)	86 (4%)	
Pedestrian	10 (1%)	16 (1%)	
Other	12 (1%)	11 (0%)	
**Neurological Level at Discharge**			<0.001
Paraplegia, incomplete	828 (64%)	1,252 (56%)	
Paraplegia, complete	360 (28%)	782 (35%)	
Paraplegia, minimal deficit	101 (8%)	187 (8%)	
Tetraplegia, incomplete	1 (0%)	3 (0%)	
**Pain Interference with Work**			<0.001
A little bit	341 (26%)	556 (25%)	
Moderately	259 (20%)	419 (19%)	
Not at all	238 (18%)	358 (16%)	
Quite a bit	177 (14%)	382 (17%)	
Not applicable (no pain)	209 (16%)	288 (13%)	
Extremely	58 (4%)	194 (9%)	
Unknown	7 (1%)	14 (1%)	
Declined	1 (0%)	8 (0%)	
Doesn't know	0 (0%)	5 (0%)	

[1]Median (Q1, Q3); n (%),[2]Wilcoxon rank sum test; Pearson's Chi-squared test

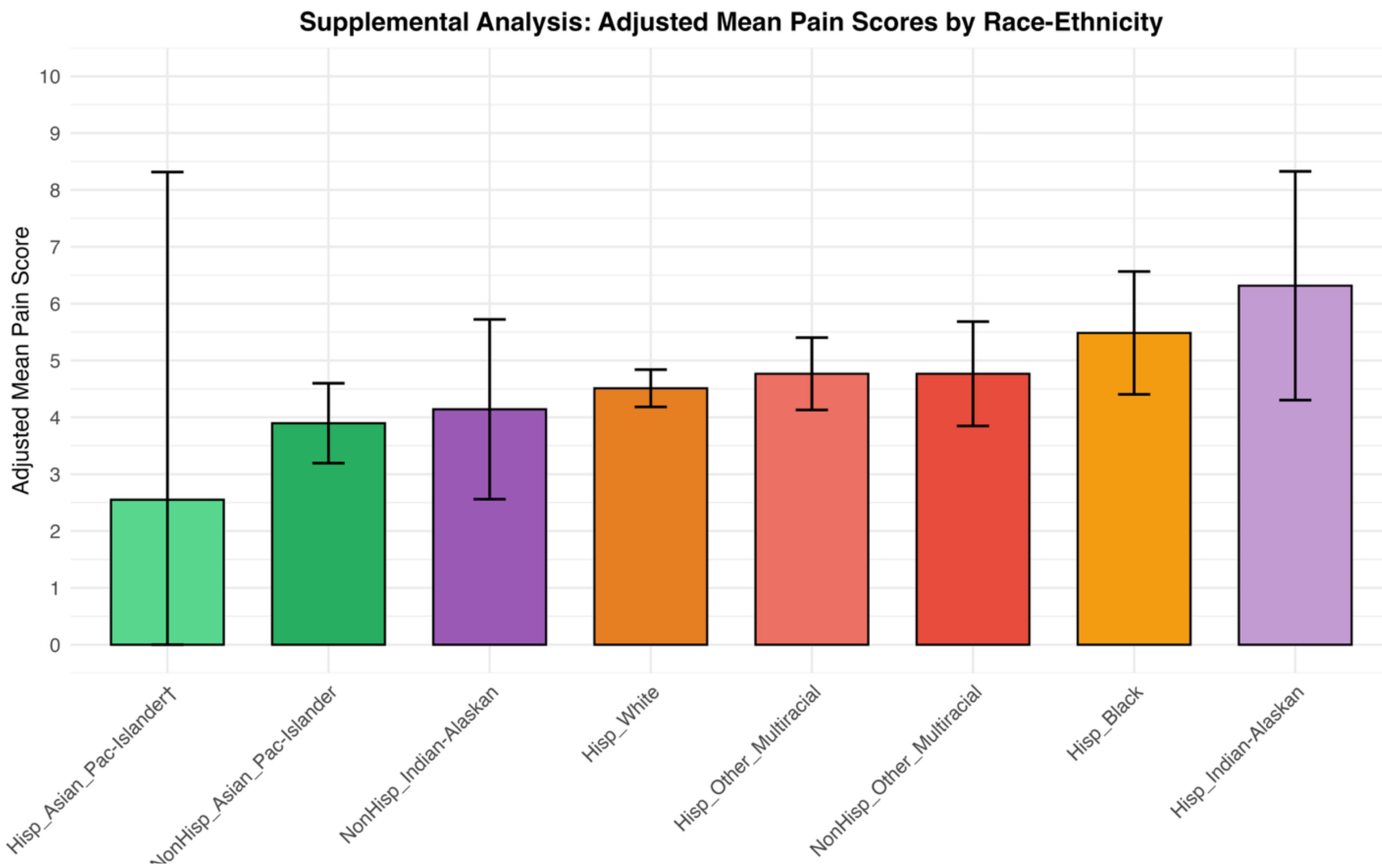

Supplemental Figure 4. Adjusted mean pain scores and pain interferences across ethnicity and races.

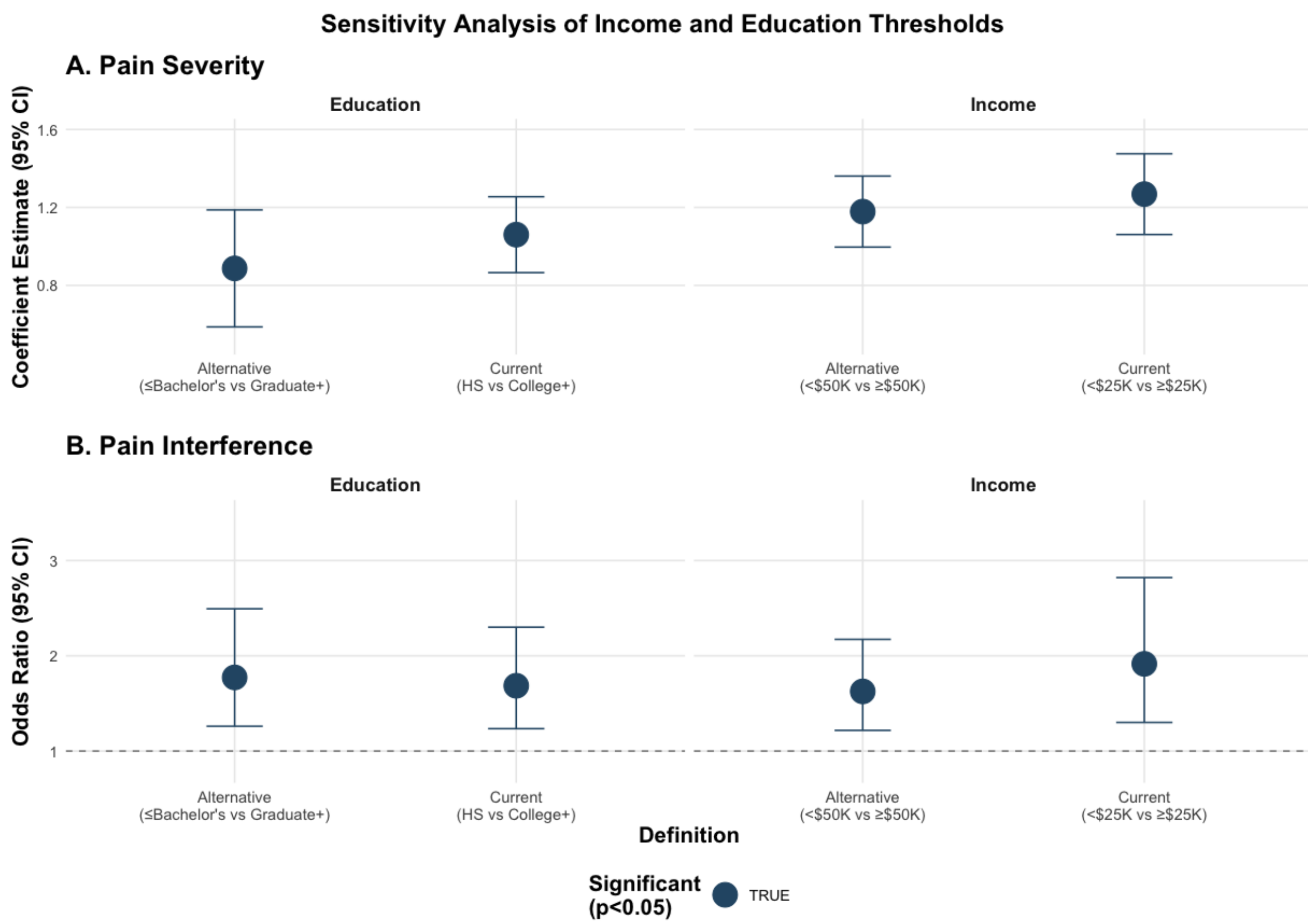

Supplemental Figure 5. Sensitivity analysis comparing alternative definitions of low income and education levels. A) Pain severity coefficient estimates and B) pain interference odds ratios and 95% confidence intervals for the association between socioeconomic status using current definitions (income: <$25,000 vs. ≥$25,000; education: high school or less vs. any college) compared to alternative definitions (income: <$50,000 vs. ≥$50,000; education: up to bachelor's vs. graduate degree).

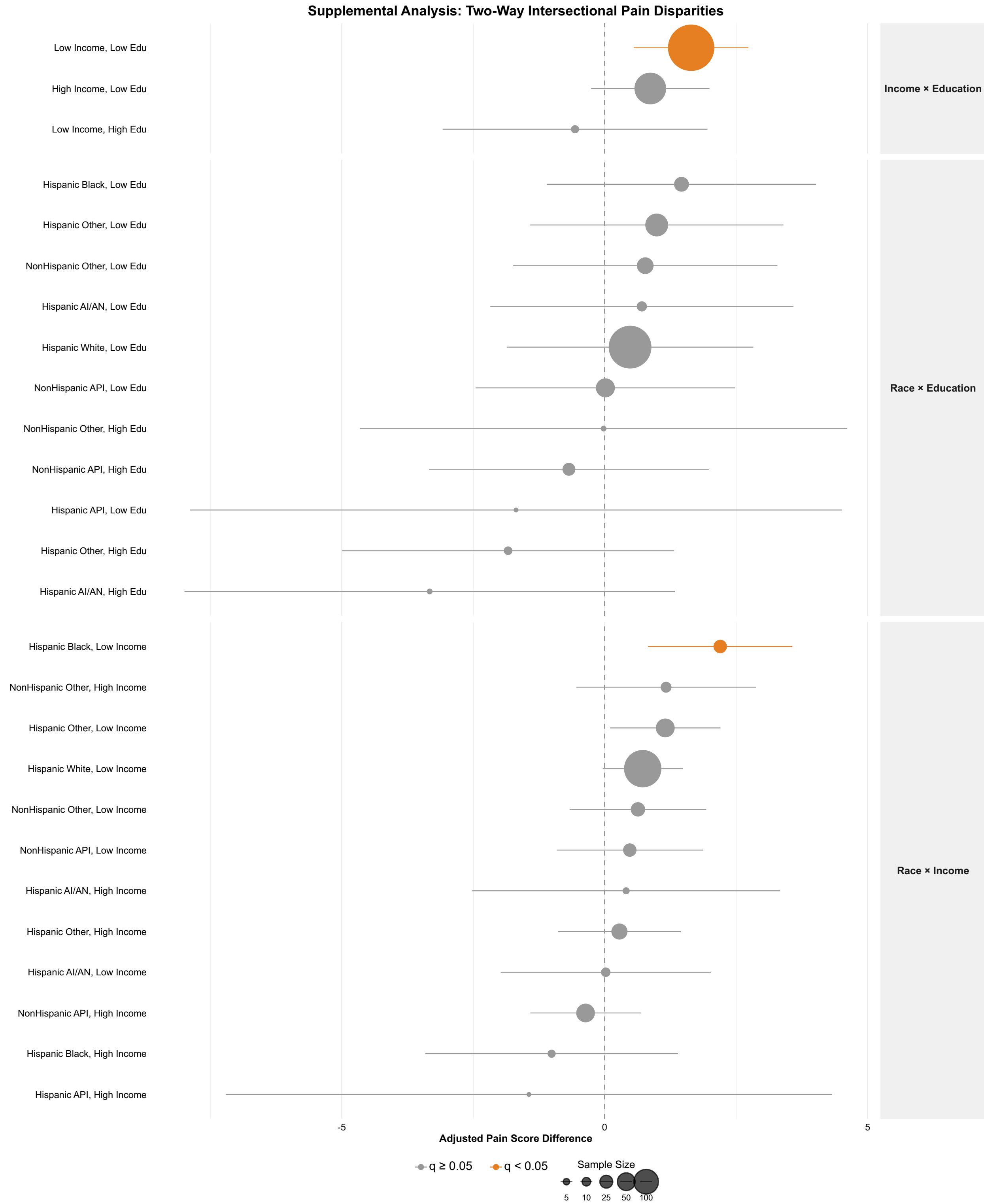

Supplemental Figure 6. Two-way intersectional pain severity scores by race/ethnicity groups contributing <10% of total sample.

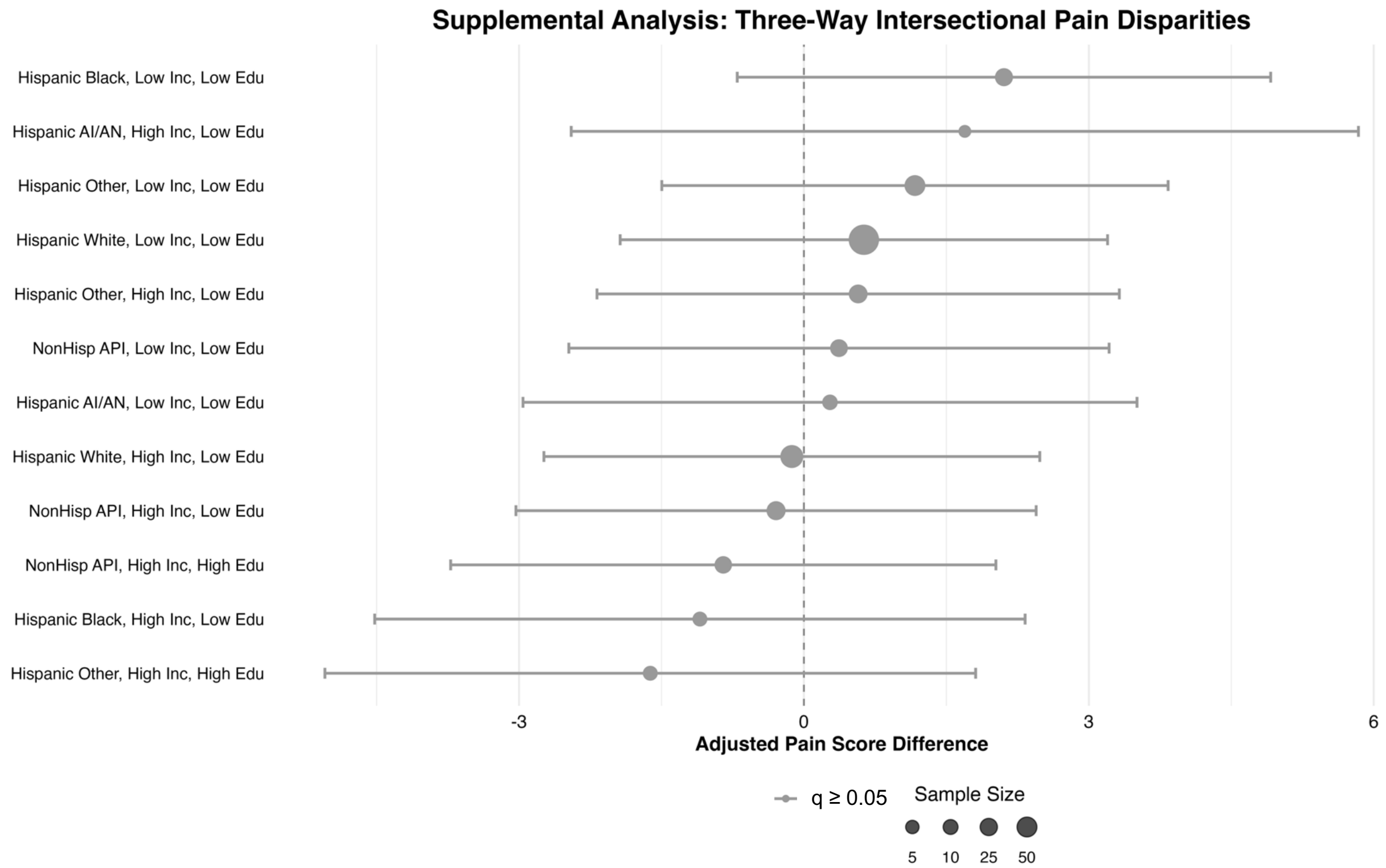

Supplemental Figure 7. Three-way intersectional pain severity scores by race/ethnicity groups contributing <10% of total sample.

Supplemental Table 7. Independent contributions of race/ethnicity, income, and education to chronic pain outcomes one year post-traumatic SCI.

Exposure	LMG Weight	95% CI	% of Explained Variance	Rank
**Pain Severity**				
Income	0.0262	0.0135–0.0377	39.4%	1
Education	0.0201	0.0102–0.0292	30.3%	2
Race/Ethnicity	0.0201	0.0095–0.0300	30.3%	3
**Pain Interference**				
Income	0.0129	0.0032–0.0215	52.0%	1
Education	0.0081	0.0012–0.0143	32.9%	2
Race/Ethnicity	0.0037	−0.0010–0.0078	15.1%	3

LMG = Lindeman-Merenda-Gold; CI = confidence interval.

Supplemental Table 8. Average percent mediated of variables identified as reaching significance.

Pain Severity					
**Groups Compared**	**Mediator**	**% Mediated (average)**	**Total N**	**Group 1 N**	**Group 0 N**
Low Education vs High Education	Days Out of House [1-Year]	15.70%	3294	2095	1199
Low Education vs High Education	CHART Mobility Score [1-Year]	22.50%	3285	2091	1194
Low Education vs High Education	Health Insurance (Private vs Non-private) [1-Year]	18.90%	3320	2109	1211
Low Education vs High Education	Perceived Health Status [1-Year]	19.30%	3303	2102	1201
Low Education vs High Education	Health Insurance (Private vs Non-private) [Acute]	16.10%	3108	1928	1180
Low Income vs High Income	Days Out of House [1-Year]	15.70%	3294	896	2398
Low Income vs High Income	CHART Mobility Score [1-Year]	20.60%	3285	895	2390
Low Income vs High Income	Health Insurance (Private vs Non-private) [1-Year]	20.30%	3320	901	2419
Low Income vs High Income	Perceived Health Status [1-Year]	23.90%	3303	892	2411
Low Income vs High Income	Health Insurance (Private vs Non-private) [Acute]	20.10%	3108	792	2316
NonHisp Black vs NonHisp White	Days Out of House [1-Year]	12.90%	2774	673	2101
NonHisp Black vs NonHisp White	CHART Mobility Score [1-Year]	20.00%	2766	671	2095
NonHisp Black vs NonHisp White	Health Insurance (Private vs Non-private) [1-Year]	17.90%	2792	676	2116
NonHisp Black vs NonHisp White	Perceived Health Status [1-Year]	18.20%	2779	676	2103
NonHisp Black vs NonHisp White	Health Insurance (Private vs Non-private) [Acute]	13.80%	2613	592	2021
**Pain Interference**					
**Groups Compared**	**Mediator**	**% Mediated (average)**	**Total N**	**Group 1 N**	**Group 0 N**
Low Education vs High Education	Alcohol Use [1-Year]	17.70%	2802	1804	998
Low Education vs High Education	Days Out of House [1-Year]	19.50%	2801	1808	993
Low Education vs High Education	CHART Mobility Score [1-Year]	26.80%	2795	1805	990
Low Education vs High Education	Health Insurance (Private vs Non-private) [1-Year]	19.00%	2813	1812	1001
Low Education vs High Education	Perceived Health Status [1-Year]	24.10%	2815	1818	997
Low Income vs High Income	Alcohol Use [1-Year]	12.30%	2802	783	2019
Low Income vs High Income	Days Out of House [1-Year]	15.70%	2801	786	2015
Low Income vs High Income	CHART Mobility Score [1-Year]	22.20%	2795	786	2009
Low Income vs High Income	Health Insurance (Private vs Non-private) [1-Year]	17.90%	2813	788	2025
Low Income vs High Income	Perceived Health Status [1-Year]	31.10%	2815	785	2030
Low Income vs High Income	Health Insurance (Private vs Non-private) [Acute]	15.60%	2619	686	1933
NonHisp Black vs NonHisp White	Alcohol Use [1-Year]	23.00%	2368	582	1786
NonHisp Black vs NonHisp White	Days Out of House [1-Year]	25.40%	2366	580	1786
NonHisp Black vs NonHisp White	Hours in Bed [1-Year]	18.50%	2370	582	1788
NonHisp Black vs NonHisp White	CHART Mobility Score [1-Year]	35.20%	2361	580	1781
NonHisp Black vs NonHisp White	Health Insurance (Private vs Non-private) [1-Year]	28.00%	2374	581	1793
NonHisp Black vs NonHisp White	Perceived Health Status [1-Year]	33.60%	2376	584	1792
NonHisp Black vs NonHisp White	Depression Severity Score [1-Year]	26.10%	1194	328	866
NonHisp Black vs NonHisp White	Health Insurance (Private vs Non-private) [Acute]	19.40%	2209	505	1704
